# Early Mesozoic burst of morphological disparity in the slow-evolving coelacanth fish lineage

**DOI:** 10.1038/s41598-023-37849-9

**Published:** 2023-07-13

**Authors:** Christophe Ferrante, Lionel Cavin

**Affiliations:** 1 Department of Geology and Palaeontology, Natural History Museum of Geneva, CP 6434, 1211 Geneva 6, Switzerland; 2 Department of Earth Sciences, University of Geneva, Rue des Maraîchers 13, 1205 Geneva, Switzerland

**Keywords:** Palaeontology, Palaeoecology

## Abstract

Since the split of the coelacanth lineage from other osteichthyans 420 million years ago, the morphological disparity of this clade has remained remarkably stable. Only few outliers with peculiar body shape stood out over the evolutionary history, but they were phylogenetically and stratigraphically independent of each other. Here, we report the discovery of a new clade of ancient latimeriid coelacanths representing a small flock of species present in the Western Tethys between 242 and 241 million years ago. Among the four species, two show highly derived anatomy. A new genus shows reversal to plesiomorphic conditions in its skull and caudal fin organisation. The new genus and its sister *Foreyia* have anatomical modules that moved from the general coelacanth Bauplau either in the same direction or in opposite direction that affect proportions of the body, opercle and fins. Comparisons with extant genetic models shows that changes of the regulatory network of the Hedgehog signal gene family may account for most of the altered anatomy. This unexpected, short and confined new clade represents the only known example of a burst of morphological disparity over the long history of coelacanths at a recovery period after the Permian–Triassic Mass Extinction.

## Introduction

Coelacanth fish, or Actinistia, have been known as fossils since the early nineteenth century and by a living species discovered along the eastern coast of Africa in 1938, followed by a second species in the western Pacific in 1998. Coelacanths are commonly referred to as ‘living fossils’ because since the Early Devonian^1^, the majority of taxa share a general body morphology that is somewhat constant over time. This conservative and monotonous morphology has been interpreted by many studies as the sign of a relatively slow rate of morphological evolution^2–5^, which is congruent with the very slow metabolism of *Latimeria* associated with its particular life history traits^6^. Exceptions to this morphologically constant general Bauplan exist in the fossil record with unusual forms appearing sporadically in the Middle-Upper Devonian, Early Carboniferous, and Triassic^7^.

**Figure 1:**
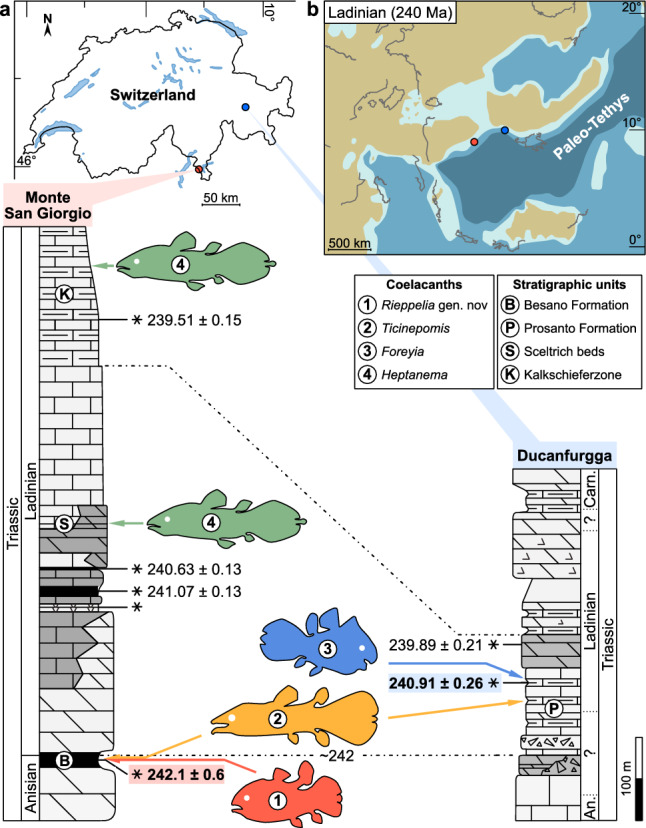
Geographical and stratigraphic location of Triassic coelacanths in Switzerland at the Monte San Giorgio and the Ducanfurgga sites, with stratigraphic correlations. (a) Map of Switzerland (designed with the software AFFINITY DESIGNER, Version 1.10.6, https://affinity.serif.com) showing location of sites (top), with coelacanth taxa placed in correlated stratigraphic sections (bottom). (**b**) Paleogeographic map (modified from Ref.^12^) showing the location of the two sections in the Ladinian (Middle Triassic).

The Paleontological Institute and Museum, University of Zurich (PIMUZ) houses fossil material from the locality of the UNESCO site of Monte San Giorgio (MSG) (Canton of Ticino, Switzerland) (Fig. 1) collected during field campaigns in the mid-twentieth century, which are studied here for the first time, except a handful of specimens studied by Olivier Rieppel in the 1980s. Rieppel described *Ticinepomis peyeri* from the Besano Formation^8^ and recorded another taxon he referred to cf. *Holophagus picenus*^9^, which corresponds to the new genus and species described below. Another species of *Ticinepomis* is present in the MSG site, as well as in the isochronous and spatially close Prosanto Formation (Canton of Graubünden)^10^. This last formation has already yielded the bizarre coelacanth *Foreyia maxkuhni*^11^.

## Systematic palaeontology

Sarcopterygii, Romer 1955

Actinistia, Cope 1871

Latimeriidae, Berg 1940

Latimeriinae subfamily nov.

### Diagnosis

Latimeriidae coelacanths characterised by the following unique combination of characters: anterior and posterior parietals of dissimilar size; supraorbito-tectal series composed of more than 10 elements; anterior branches of supratemporal commissure present; dermal bones of the skull mostly or entirely unornamented; infraorbital canal running along the anterior margin of the postorbital; anterior and/or posterior branches of the infraorbital canal present; jugal sensory canal running along the ventral margin of the squamosal; coronoid opposite to the posterior end of dentary modified; toothed area of the parasphenoid restricted to the anterior half.

Ticinepomiinae subfamily nov.

### Diagnosis

Latimeriidae coelacanths characterised by the following unique combination of characters: anterior and posterior parietals of similar length; supraorbitals as wide as parietals; posterior margin of the skull roof straight; preorbital present; postorbital reduced to a narrow tube surrounding the sensory canal only; lachrymojugal more or less thick, triangular in shape; splenial with an anterior portion curved downward; splenial forming a symphyseal pore; medial branch of the otic canal on the postparietal absent; short body with less than 50 neural arches; ossified lung absent; lobe of the pectoral fin poorly developed; supplementary caudal lobe enclosed in the caudal fin profile; denticles on the fin rays of the anterior dorsal fin and the caudal fin.

*Rieppelia heinzfurreri* gen. et sp. nov.

### Etymology

The generic name honours Dr Olivier Rieppel who was the first to mention the presence of this coelacanth taxon in the fauna of Monte San Giorgio. The specific name honours Dr Heinz Furrer for his important contribution on the geology and palaeontology of Monte San Giorgio and the Triassic of Switzerland.

### Holotype

**Figure 2:**
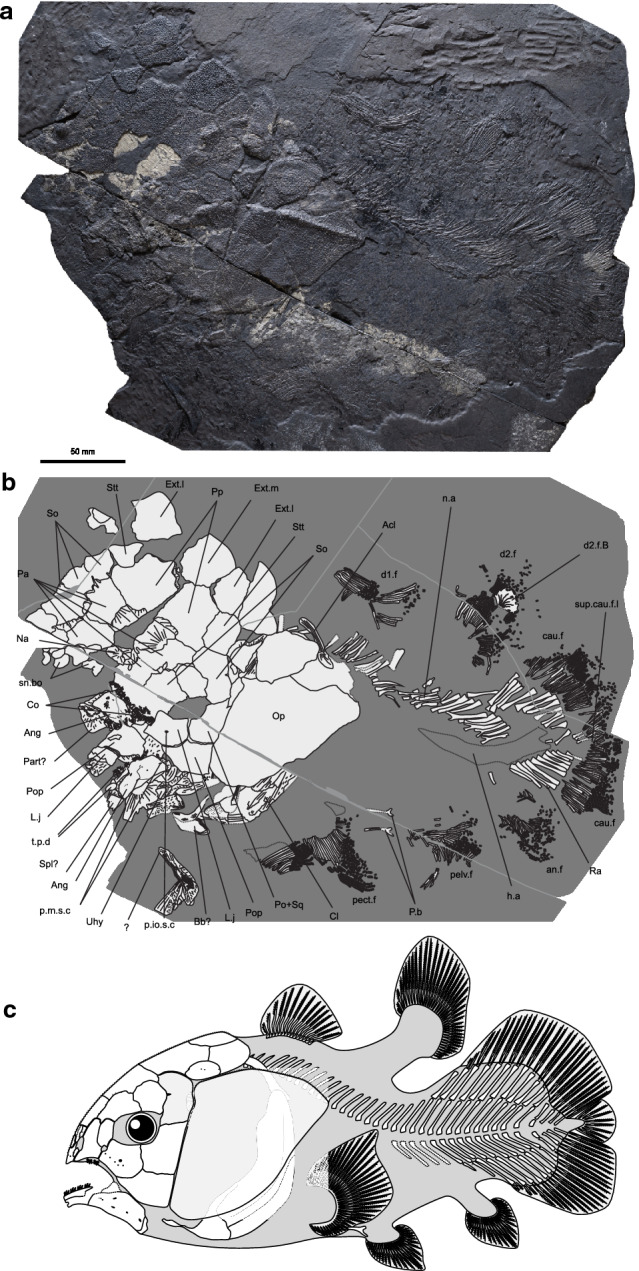
Skeleton of *Rieppelia heinzfurreri* gen. et sp. nov. (**a**) Photograph and (**b**) outline of the holotype (PIMUZ T 5902). (**c**) Reconstruction of the whole skeleton. *Acl* anocleithrum, *ana.f* anal fin, *Ang* angular, *Bb* basibranchial, *cau.f* caudal fin, *Cl* cleithrum, *Co* coronoid, *d1.f* anterior dorsal fin, *d2.f* posterior dorsal fin, *d2.f.B* basal plate of the posterior dorsal fin, *Ext.l* lateral extrascapular, *Ext.m* median extrascapular, *h.a* haemal arches, *L.j* Lachrymojugal, *n.a* neural arches, *Na* nasal, *Op* opercle, *P.b* pelvic bone, *p.io.s.c* pore for the infraorbital sensory canal, *p.m.s.c* pore for the mandibular sensory canal, *Pa* parietal, *Part* prearticular, *pect.f* pectoral fin, *pelv.f* pelvic fin, *Po* + *Sq* postorbital + squamosal, *Pop* preopercle, *Pp* postparietal, *Ra* radial, *sn.bo* snout bones, *So* supraorbital, *Spl* splenial, *Stt* supratemporal, *sup.cau.f.l* supplementary caudal fin lobe, *t.p.d* tooth plate, *Uhy* urohyal.

PIMUZ T 5902, complete specimen of 300 mm total length in left lateral view (Fig. 2a,b).

### Paratypes

**Figure 3:**
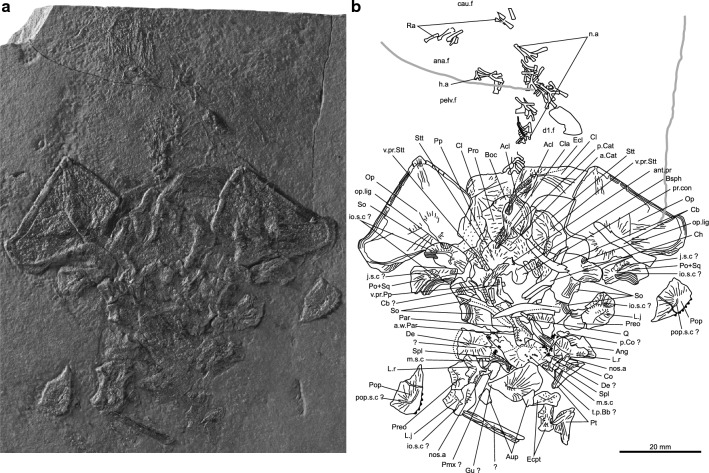
Juvenile or newborn specimen of *Rieppelia heinzfurreri* gen. et sp. nov. (**a**) Photograph and (**b**) outline of the paratype PIMUZ T 3376 showing bones of the skull in internal view and the axial skeleton. *a.Cat* anterior catazygal, *a.w.Par* ascending wing of parasphenoid, *Acl* anocleithrum, *ana.f* anal fin, *Ang* angular, *ant.pr* antotic process, *Aup* autopalatine, *Boc* basioccipital, *Bsph* basisphenoid, *cau.f* caudal fin, *Cb* ceratobranchial, *Ch* ceratohyal, *Cl* cleithrum, *Cla* clavicle, *Co* coronoid, *d1.f* anterior dorsal fin, *d2.f* posterior dorsal fin, *De* Dentary, *Ecl* extracleithrum, *Ecpt* ectopterygoid, *h.a* haemal arches, *io.s.c* infraorbital sensory canal, *j.s.c* jugal sensory canal, *L.j* lachrymojugal, *L.r* lateral rostral, *m.s.c* mandibular sensory canal, *n.a* neural arches, *nos.a* anterior nostril, *nos.p* posterior nostril, *Op* opercle, *op.lig* insertion point for opercular ligament, *p.Cat* posterior catazygal, *p.Co* principal coronoid, *Par* parasphenoid, *pelv.f* pelvic fin, *Pmx* premaxilla, *Po* + *Sq* postorbital + squamosal, *Pop* preopercle, *pop.s.c* preopercular sensory canal, *Pp* postparietal, *pr.con* processus connectens, *Preo* preorbital, *Pro* prootic, *Pt* pterygoid, *Q* quadrate, *Ra* radial, *So* supraorbital, *Spl* splenial, *Stt* supratemporal, *t.p.Bb* basibranchial tooth plate, *v.pr.Pp* ventral descending process of the postparietal, *v.pr.Stt* ventral descending process of supratemporal.

PIMUZ T 1638 (Fig. S1), almost complete skull roof of 105 mm long in internal view on part and counterpart; PIMUZ T 3376 (Fig. 3), subcomplete juvenile/newborn specimen of circa 100 mm long with the entire skull (50 mm long) in internal view; PIMUZ T 5905 (Fig. S2), complete skull roof of 132 mm long in external view.

### Referred material

39 specimens mostly represented by scattered bones (a complete list is provided in the Supplementary Information).

### Locality and horizon

Monte San Giorgio (Canton of Ticino, Switzerland; Province of Varese, Italy); Besano Formation, Middle and Upper Members, *Nevadites secedensis* Ammonoid Zone, Late Anisian (Middle Triassic).

### Diagnosis

Ticinepomiinae coelacanth characterised by the following unique combination of characters: intracranial joint sutured; cheek bones sutured; independent squamosal absent; lachrymojugal short and deep; anterodorsal excavation of the postorbital absent; lateral rostral dorsally enlarged, separating the unique tectal from the supraorbital series; opercle hypertrophied; point of attachment for the opercular ligament on the supraorbital; cleithrum hypertrophied with a large branchial lamina; coronoid composed of a cluster of three tiny curved and pointed teeth; dentary tooth plates bearing numerous tiny curved and pointed teeth; urohyal short and ovoid shaped; posterior dorsal and pectoral fins hypertrophied; caudal fin with a one-to-two ratio between radials and fin rays; scales subcircular-to-suboval with an exposed area ornamented with numerous blunt spines.

### Measurements and meristic

Estimated total body length: 630 mm; d1.f = 15; d2.f = 38–46; pect.f = 37–44; pelv.f = 30; ana.f = 30; cau.f = 30/24; n.a = 35; h.a ~ 14–16.

### Nomenclatural act

The present work and its nomenclatural act are registered in ZooBank, the online registration system for the International Commission on Zoological Nomenclature. The Life Science Identifiers for this publication is “urn:lsid:zoobank.org:pub:5D84D650-A188-4B91-888B-87D28A445910” and can be resolved appending the prefix “http://zoobank.org/” in any standard web browser.

## Description

*Rieppelia* is a distinctively short coelacanth. The length of the head (without the enlarged opercle), the trunk and the caudal fin are each one third of the total body length, giving *Rieppelia* a plump appearance (Fig. 2) shared with *Foreyia*^11^.

The skull roof of *Rieppelia* departs from the standard morphology of latimerioids coelacanths by its parietonasal shield being approximatively the same length than the postparietal shield, a feature commonly found in Palaeozoic coelacanths. All the bones making up the skull roof—except the snout bones—are tightly sutured to one another. Parietonasal and postparietal shields are in close contact, and when a gap is visible for taphonomic reasons the postparietals show areas of overlaps for suturing with the posterior parietals (Fig. S3a–c), indicating that both shields were strongly sutured together and the intracranial joint not functionnal. This derived character shared with *Foreyia*^11^ is unique among coelacanths.

Beside the unusual morphology of some dermal bones, the skull roof pattern of *Rieppelia* is characteristic of coelacanths (Fig. 2, Supplementary Figs. S1, S2, S3a,b). The snout is composed of rostral ossicles and a pair of premaxillae carrying a dorsal lamina not perforated for the anterior opening of the rostral organ, which opens laterally to the lamina. The lateral series of the parietonasal shield is composed of a single small tectal and three supraorbitals. The supraorbitals are as wide as the parietals, like in *Foreyia* and *Ticinepomis* and unlike the Latimeriinae. The tectal does not contacts the supraorbital series because the enlarged dorsal portion of the lateral rostral separates them. The preorbital is not perforated by the posterior openings for the rostral organ that supposedly open in the anterior part of the orbital space, in a position similar to that of *Latimeria*. The median series is composed of one pair of nasals, sometimes separated by an internasal, and two pairs of parietals. The nasals are considerably smaller than the parietals. The two pairs of parietals are of similar size, as in *Ticinepomis* and *Foreyia*, unlike in the Latimeriinae. The postparietals are large bones each flanked by a smaller supratemporal. Ventral descending processes are present on the posterior parietals, the postparietals and the supratemporals. The extrascapular series includes a median extrascapular flanked on both side by a lateral extrascapular and occupies half the area of the postparietal shield, each extrascapular being as large as a postparietal. Extrascapulars hypertrophied to such a degree are unique among coelacanths, only possibly shared with *Foreyia* in which all the postparietal shield bones are fused together and form a hypertrophied dome^11^.

The cheek bones (Figs. 2a,b, 3, Supplementary Fig. S3a,b) show areas of mutual overlap and are tightly sutured to each other, which is a unique condition among post-Palaeozoic coelacanths. At the posterior margin of the orbit is a large bone interpreted as the fusion of the postorbital and the squamosal. The lachrymojugal is short and thick and roughly triangular in shape, reminiscent of the lachrymojugal of *Foreyia* (lachrymojugal + squamosal in Ref.^11^) and, to a lesser extent, of *Ticinepomis*^10^. At the junction of the lachrymojugal, preorbital and lateral rostral, the bones are notched and form the outlet of the posterior nostril. The anterior nostril opens below the lateral rostral. The opercle (Figs. 2, 3, Supplementary Figs. S1a,b, S3a,b, S4), ovoid-to-triangular shaped, is hypertrophied, covering a greater area than the whole surface formed by the cheek and supraorbitals together. The antero-dorsal corner of the opercle produced as a small ridged process which faces a similar ridged surface on the posterior-most supraorbital, providing points of attachment for the opercular ligament.

The lower jaw is small but with an organisation comparable to that of other coelacanths. However, the dentition (Figs. 2, 3, Supplementary Figs. S1c, S5a) is very distinctive with many small, pointed, curved teeth, either in packs borne by dentary tooth plates or in clusters of three teeth corresponding to coronoids and dermopalatine. The symphyseal margin of the splenial is notched such that it forms a large symphyseal pore upon contact with its antimere. This character is shared with a few other coelacanths, notably *Ticinepomis* and *Foreyia*.

All of the dermal bones—except the gular plates, the oral margin of the premaxillae and the bones of the pectoral girdles—are heavily ornamented with numerous distinct rounded and pointed odontodes (Fig. S6), an ornamentation similar to that of *Foreyia*.

The cleithrum is broad and boomerang-shaped with an enlarged anterolateral lamina compared to other coelacanths (Figs. 2, 3, Supplementary Fig. S4). The pectoral and the posterior dorsal fins are overdeveloped compared to the pelvic and anal fins. The first anterior dorsal fin has a high number of rays with 15 rays, as in *Foreyia*^11^, but the rays are however shorter than in *Foreyia*. The axial skeleton is very short, composed of 35 neural and about 15 haemal arches. This low number of neural arches, identical to that of *Foreyia*, is one of the lowest known among coelacanths but is not related to body size as *Rieppelia* is twice as large as *Foreyia*. In the caudal fin, rays outnumber the radial supports by two-to-one, a ratio known only in Palaeozoic coelacanths. The caudal fin has a rounded posterior contour that encloses the supplementary lobe, reminiscent of the caudal fin of *Foreyia*^11^. The scales and all fin rays are ornamented with blunt denticles (Fig. S7). A more complete description of *Rieppelia* is provided in the Supplementary Information.

## Phylogenetic analysis

In order to appraise the systematic position of *Rieppelia*, we ran a maximum parsimony analysis based on a list of characters modified from the analysis of Forey^7^. Compared to the last version of this list by Toriño et al.^13^, we removed 16 characters, modified 15 other characters definitions and added 18 new characters, resulting in a list of 112 characters. We found inconsistencies between the scorings and available descriptions for 37 taxa, and consequently corrected 170 character states. Our data matrix includes 46 ingroup taxa and one outgroup taxon. We removed *Luopingcoelacanthus* and *Yunnancoelacanthus* because they present too many uncertainties and discrepancies. The systematic status of *Styloichthys* as a coelacanth is still debated^14^ and we preferred to remove this taxon from our analysis. We used *Onychodus jandemarrai* described by Andrews et al.^15^ as a new outgroup. The character list and data matrix are provided in the Supplementary Information.

**Figure 4:**
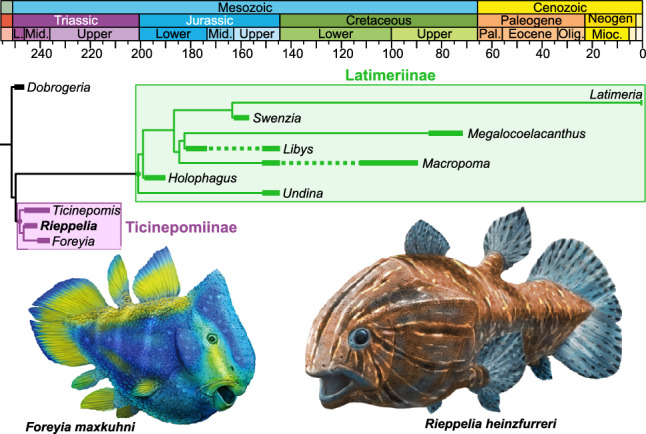
Time-tree phylogeny of Latimeriidae, plotted against the International Chronostratigraphic Chart, with reconstructions of *Foreyia* and *Rieppelia* gen. nov. Artwork by Alain Bénéteau.

After running our phylogenetical analysis, we obtained 135 most parsimonious trees of 310 steps with a consistency index of 0.397, a retention index of 0.701 and a rescaled consistency index of 0.278. The strict consensus tree resolves *Rieppelia* as sister to *Foreyia*, with *Ticinepomis* as sister genus to the pair (Fig. S9a,b). The three genera form the subfamily Ticinepomiinae nov., which is sister to the Latimeriinae nov. Both subfamilies are among the best supported clades of our phylogeny and both, plus the sister genus *Dobrogeria*, form together the Latimeriidae (Fig. 4).

## Discussion

The ticinopomiins are a newly recognised clade present within a very short time span, approximately one million year, containing three genera, *Ticinepomis, Rieppelia* and *Foreyia* (Fig. S9c), the two latter with a highly derived morphology. An indeterminate coelacanth thought to be a relative of *Foreyia* found in a slightly older locality from Northern Dolomites, Italy, possibly belong to this clade^16^. Except for the latter, Ticinepomiins were found in the Prosanto and Besano Formations, which were deposited at the westernmost end of the Paleotethys in poorly connected shallow intraplatform lagoons or basins due to the presence of carbonate platforms^10,17^ (Fig. 1b). These environments were favourable to endemism, especially for organisms with low dispersal capacity. Most of the biogeographical patterns detected in the evolutionary history of coelacanths are best explained by allopatric speciation associated with plate tectonic-related vicariant or dispersal events^18^, but ticinepomiin diversification is an exception probably corresponding to sympatric speciation. This small burst of taxic and morphological diversification is remarkable when placed in the lineage of the slow-evolving, morphologically monotonous coelacanths, though in no way comparable in intensity to species flocks that may affect some teleost clades^19,20^.

The relatively confined environment was favourable to speciation, with the production of specialist niches characterised by morphologically original forms, especially when replaced within the larger pattern of recovery of life still occurring 10 million years after the Permian–Triassic Mass Extinction (PTME)^21^. The occupation of new ecological niches by bony fish after the PTME, resulting in particular in new modes of predation and defence, heralds the ongoing Mesozoic Marine Revolution in the Middle Triassic^22^. The recent discovery of the Guiyang Biota in southern China, dated about a million years after PTME, indicates that this revolution may have started earlier, shortly after the mass extinction^23^. Interestingly, the Guiyang Biota consists of a complex trophic network, in which two large coelacanths occupied the position of apex predators^23^. This Early-Middle Triassic diversification also affected basal neopterygians, with specialisations in their feeding apparatus^24^, and produced deep-bodied forms^25,26^. The morphological characteristics of *Foreyia* and *Rieppelia* are comparable to the latter forms, although they occupied a slightly different region of the morphospace than contemporary deep-bodied ray-finned fishes due to their strong modification of the anatomy of the skull. Ticinepomiins exemplified a new body shape pattern for the bony fishes of this time interval that lasted a very short time on the geological scale.

If the environment is at the origin of selective pressures necessary for sympatric speciation, the developmental genetic machinery of the ticinepomiins must still be able to ensure the phenotypic variability necessary for the action of selection. The discovery of *Foreyia* led to a search for potential genes involved in altered cranial and postcranial anatomy^11^. The discovery of *Rieppelia*, the sister genus of *Foreyia*, makes it possible to continue this research.

**Figure 5:**
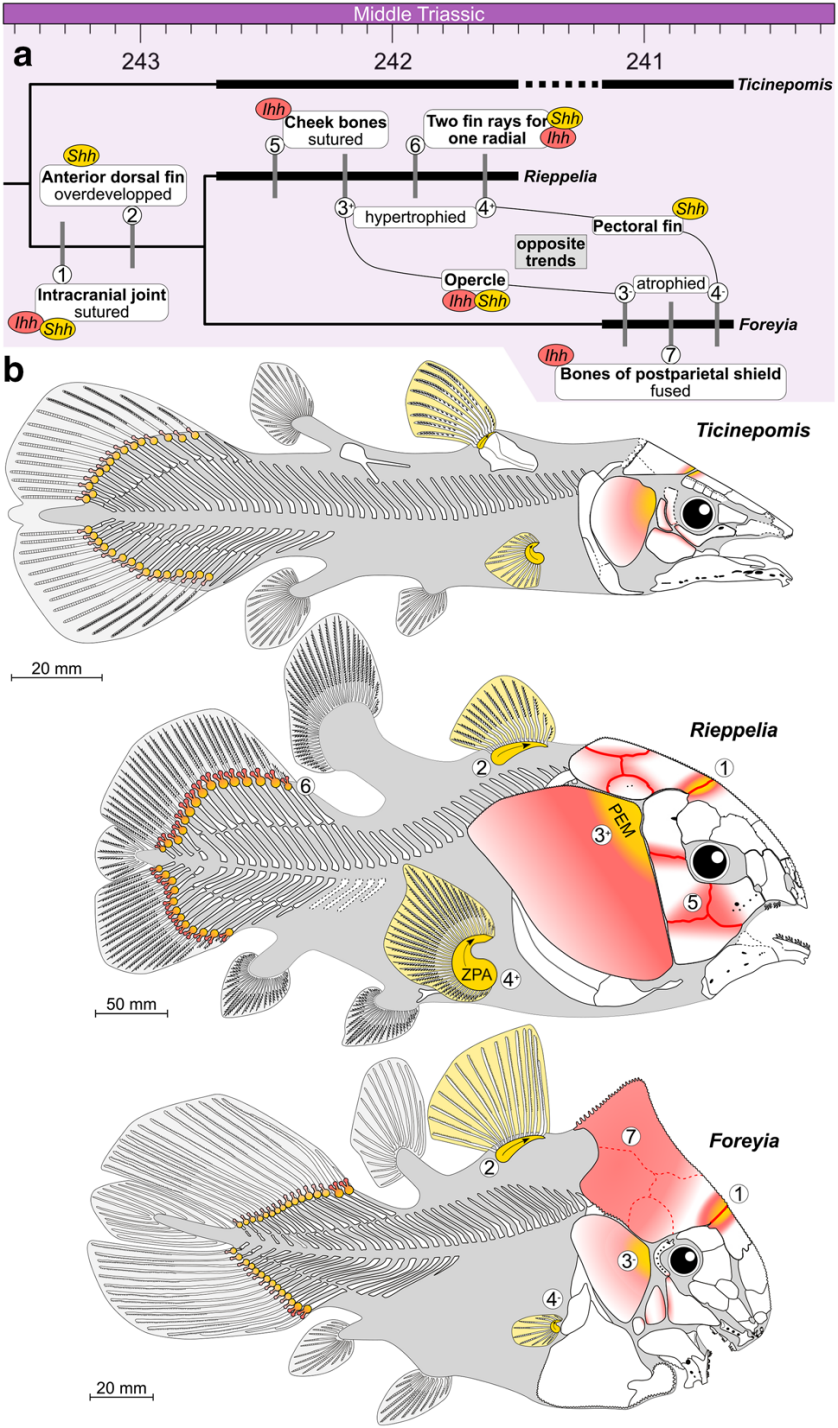
Hypothetical impacts of changes in the regulatory system of two Hedgehog genes that could explain most of the altered anatomy of the *Foreyia* and *Rieppelia* gen. nov. (**a**) Presumed anatomical changes in relation to genetic regulation of Sonic (*Shh*) and Indian (*Ihh*) hedgehog genes that may account for altered morphology, in similar or opposite direction, mapped onto a time-tree phylogeny of Ticinepomiinae. (**b**) Morphological modules potentially affected by changes in the regulatory system of *Shh* and *Ihh* (built on comparisons with extant vertebrate models). *PEM* posterior ectodermal margin, *ZPA* zone of polarizing activity. Drawings of *Ticinepomis* and *Foreyia* modified from Refs.^10,11^, respectively.

The three genera of ticinepomiins deviate from the general Bauplan coelacanth mainly by changes in size proportions of anatomical modules and changes in meristic features, especially pronounced in *Foreyia* and *Rieppelia* (Fig. 5). The anatomical shifts in the same direction in *Foreyia* and *Rieppelia* are their proportionally enlarged head and tail coupled with their shortened vertebral column, the sutured intracranial joint and a high number of rays in the first dorsal fin. The anatomical changes in opposite directions in both genera are the number of pectoral fin rays, reduced in *Foreyia* and over numbered in *Rieppelia*, and the size of the opercle compared to *Ticinepomis* (and *Latimeria*), moderately reduced in *Foreyia* and hypertrophied in *Rieppelia*. It should be noted that *Latimeria* has an unmineralised opercular flap^7^, the surface of which is approximately comparable to the mineralised enlarged opercle of *Rieppelia*. All ticinepomiins are also characterised by reversal to plesiomorphic conditions present in basal actinistians, namely the presence of a preorbital and the presence of a series of short rays in front of the first dorsal fin. Finally, *Rieppelia* is characterised by supplementary reversals to plesiomorphic conditions, namely the suturing of its cheek bones and the presence of two fin rays per radial in the caudal fin, and *Foreyia* is characterised by the fusion of the bones of its postparietal shield.

Since the morphological changes affect similar morphological modules in *Foreyia* and *Rieppelia*, we hypothesise that the relevant genetic mechanism more likely involves the gene regulation network, a process that has long been considered a potential cause of “bursts of evolutionary advances”^27^.

Ticinepomiin genomes are obviously not accessible, but information from *Latimeria* and phylogenetic bracketing vertebrate models (tetrapods and teleosts) provides clues to identify possible genetic mechanisms underlying the altered anatomy of *Foreyia* and *Rieppelia*. Here we focus on the Hedgehog (*Hh*) signal gene family that controls many critical steps of cell differentiation and patterning during development, in part because it is known to play an important role in vertebrate skeletal morphogenesis, and in part because its genetic pathways and regulatory network are well known in several clades of vertebrates^28–30^. We do not assume here that *Hh* activity was necessarily involved in the evolutionary process of ticinepomiins, but we have chosen this gene family as a model to evaluate the potential impact of the regulatory mechanism of a morphogen on the observed ticinepomiin phenotypes. The Indian hedgehog (*Ihh*) plays an important role in the skeletal differentiation of endochondral bones, but also of cranial dermal bones^31–33^, as well as in fin ray bifurcation^34^. It functions as an enhancer of opercle development in teleosts, as shown in zebrafish *ihha*^*-*^ mutant in which the opercle and subopercle are fused^35,36^. Indeed, the enlarged opercle of *Rieppelia* covers the area normally occupied by the opercle and subopercle in other coelacanths, and a fusion of these two bones cannot be excluded during ontogenesis in the genus. Sonic hedgehog (*Shh*), which functions as a morphogen, mitogen and differentiation regulator^37^, is active in jawed vertebrates in an epithelial signalling center called the posterior ectodermal margin (PEM), which acts on the underlying mesenchyme that generates the opercle^38^ and is a potential proliferative driver of the posterior expansion of the second pharyngeal arch in the chick embryo and in the zebrafish which eventually extends posteriorly to form the opercle^39^. This gene plays roles in the craniofacial development^37,40–43^, but also in the postcranial skeleton, particularly in ray bifurcation^35,44^. The suppression of a repressor of *Hh* signaling (*Gli3*) leads to craniosynostosis of the lambdoid suture in mice^33,45^. This suture is considered homologous to the intracranial joint in coelacanths (with the addition of a neoformation, the posterior parietal intercalated between the anterior parietal and the postparietal)^42^, which indeed shows a craniosynostosis in *Foreyia* and *Rieppelia*. The Shh glycoprotein, secreted from the zone of polarising activity (ZPA) in tetrapods and teleosts^37,44,46^, and expressed in the dorsal fin of teleosts^46^, plays a role in branching morphogenesis mechanism in the zebrafish^44^. Regulatory mutations of the *Shh* gene can cause preaxial polydactyly in tetrapods^47,48^.

The cis-regulatory module of *Shh* in *Latimeria* is unique among vertebrates in that it contains midline enhancers that are absent in either actinopterygians or tetrapods, and therefore likely represents ancestral vertebrates set of midline enhancers^28^. Lang et al.^28^ considered the conserved cis-regulatory architecture of this set of *Shh* enhancers to be consistent with slow evolutionary rate of *Latimeria*. The full set of enhancers in the cis-regulatory module may also have underpinned the phenotypic variations observed in *Foreyia* and *Riepplia*.

This brief overview indicates that altered regulation of the *Hh* gene family can underlie heterochrony, an important source of evolutionary changes. Morphological features common to *Foreyia* and *Riepplia* potentially caused by heterochronic shift of the onset of expression of *Hh* family genes are the fusion of the intracranial joint and the enlarged first dorsal fin, potentially under the control of *Ihh* and *Shh* for the first, and *Shh* for the second. Changes in regulation of these genes may also have caused the atrophied *vs* hypertrophied opercles and pectoral fins of *Foreyia* and *Rieppelia* via the PEM and ZPA centers, respectively. Eventually, derived characters of *Rieppelia*, such as the sutured cheek bones and the caudal fin rays bifurcation may have also been caused by variations in the *Ihh* regulation. Why such a morphologically remarkable diversification occurred only once during the coelacanth's 420 million year evolutionary history, however, remains an open question.

## Methods

### Provenance and preparation of the specimens

The specimens were collected from the UNESCO site of Monte San Giorgio (Fig. 1a) during systematic excavations carried out by the Paleontological Institute and Museum of the University of Zurich (PIMUZ) and during sporadic field trips between 1923 and 1978. Most of the fossils, 34, were found in the Swiss locality of Meride (Canton of Ticino, Switzerland) and few others, 5, in the Italian localities of Porto Ceresio and Besano (Province of Varese, Italy).

Among these 39 specimens, 8 specimens have been selected for more preparation, which was carried out by one of us (CF) and by three preparators from the MHNG, PIMUZ and the JURASSICA Museum (Porrentruy, Canton Jura, Switzerland). The very fragile fossil material preserved on slabs of black bituminous shales were mechanically prepared using various methods. Final preparation was done under the microscope using a sand-blaster, with sodium bicarbonate used as abrasive (harder abrasives were too destructive to the fossils). In order to obtain the optimal effect, pressure and the quantity of grains ejected per second were modulated according to the hardness of the matrix and the fossil. However, a very low pressure ranging between 0.5 and 1.5 bars was mainly used. Few specimens, those preserved on bright dolomitic layer, were hard enough to be prepared using pneumatic tool, and eventually finished with sand-blasting.

A specimen (PIMUZ T 5905) was scanned with the microtomography beamline at the European Synchrotron Radiation Facility in June 2022 (10.15151/ESRF-ES-788657609). Although not yet segmented, preliminary image analysis shows that no endochondral bone of the neurocranium is preserved, contrary to our expectation, and the 3D rendering of this imaging technique will likely yield little new information.

### Thin sections

The thin sections were made and described in a previous study^49^, in which the method of confection of thin sections is detailed.

## Supplementary Information

Supplementary Information.

## Data Availability

The holotype (PIMUZ T 5902), paratypes (PIMUZ T 1638; PIMUZ T 3376; PIMUZ T 5905) and all other specimens of *Rieppelia heinzfurreri* used in this study (including those listed in the ‘Supplementary Information’) are kept in the collection of the Paläontologisches Institut und Museum der Universität Zürich (Canton Zürich, Switzerland). The protocols used in the development of this study are available in the ‘Supplementary Information’.
