# Supplementary material for: Early Mesozoic burst of morphological disparity in the slow-evolving coelacanth fish lineage
Source: Sci Rep. 2023 Jul 13;13:11356. doi: 10.1038/s41598-023-37849-9 (PMC10345187; doi:10.1038/s41598-023-37849-9)
Supplement: Supplementary file 1 — Supplementary Information. [file 41598_2023_37849_MOESM1_ESM.pdf]

Supplementary Information for:

Early Mesozoic burst of morphological disparity in the slow-evolving coelacanth fish lineage

Christophe Ferrante*[1,2] & Lionel Cavin[1]

[1]Department of Geology and Palaeontology, Natural History Museum of Geneva, CP 6434, 1211 Genève 6, Switzerland (*paleo-ferrante.ch@bluewin.ch)

[2]Department of Earth Sciences, University of Geneva, Rue des Maraîchers 13, 1205 Genève, Switzerland

Table of Contents

1 LIST OF MATERIAL 3

2 DETAILED DESCRIPTION OF *RIEPPELIA HEINZFURRERI* GEN. ET SP. NOV. 4

2.1 General morphology 4

2.2 Dermal bones of the skull roof 4
2.2.1 Parietonasal shield 5
2.2.2 Postparietal shield 8

2.3 Dermal bones of the cheek 9

2.4 Lower jaw 13

2.5 Sensory canals, cranial nerves and pit lines 15
2.5.1 Sensory canals 16
2.5.2 Otic lateral line nerve 18
2.5.3 Pit lines 20

2.6 Neurocranium and parasphenoid 20

2.7 Palatoquadrate, hyoid and gill arches 22
2.7.1 Palatoquadrate 22
2.7.2 Branchial arches and urohyal 23
2.7.3 Hyoid arch 24

2.8 Postcranial skeleton 24
2.8.1 Axial skeleton 24
2.8.2 Unpaired fins 25
2.8.3 Pectoral girdle and fin 27
2.8.4 Pelvic girdle and fin 28

2.9 Ornamentation and histology 28
2.9.1 Dermal bones 28
2.9.2 Scales 30

3 SUPPLEMENTARY FIGURES 32

4 PHYLOGENETICAL ANALYSES 47

4.1 List of characters 47

4.2 Datamatrix 63

5 REFERENCES 64

1 List of material

PIMUZ T 23, bed 115, (S); **27**, bed 132, (S); **31**, bed 115, (S); **1271**, bed 118, (S); **1273**, bed 120, (S); **1288**, bed 118, (S); **1321**, bed 118, (S); **1325**, bed 118, (S); **1357,** bed 113, (S); **1360**, bed 114, (S); **1458**, bed 104, (S); **1631**, bed 138, (I); **1632**, bed 115, (S); **1633**, bed 107, (S); **1634**, bed unknown, (I); **1635**, bed unknown, (S); **1636**, bed unknown, (S); **1638**, bed 84, (S); **1639**, bed 131, (S); **1640**, bed unknown, (S); **1755**, bed 115, (S); **1777**, bed 72, (S); **1800**, bed 116, (S); **1801**, bed 114, (S); **2558**, bed 138, (S); **3376**, bed unknown, (S); **4013**, bed 131, (S); **4936**, bed unknown, (S); **4985**, bed 142, (S); **5299**, bed 82, (S); **5320,** bed 113, (S); **5540**, bed 95, (S); **5900**, bed 73, (S); **5902**, bed 115, (S); **5903**, bed 131, (S); **5904**, bed 115, (S); **5905**, bed 132, (S); **5906**, bed unknown, (I); **5907**, bed 116, (S); all from the Ladinian (Middle Triassic), Middle and Upper Members of the Besano Formation, Switzerland (S) and Italy (I).

2 Detailed description of *Rieppelia heinzfurreri* gen. et sp. nov.

2.1 General morphology

Rieppelia is a medium size coelacanth than can reach an estimated total body length of about 630 mm, based of the largest skull (PIMUZ T 1755). The largest articulated specimen (PIMUZ T 4985) measures about 440 mm. The holotype (PIMUZ T 5902), measuring 300 mm long, therefore corresponds to an individual of intermediate size. The smallest articulated specimen (PIMUZ T 3376) has a body length of 100 mm and represents a juvenile or newborn individual as indicated by its proportionally long head measuring the half of the total body length.

Rieppelia is a distinctively short coelacanth. The length of the head (without the enlarged opercle), the trunk and the caudal fin each represent one third of the total body length, giving *Rieppelia* a plump appearance shared with *Foreyia*[1].

2.2 Dermal bones of the skull roof

Dermal bones of the skull roof of *Rieppelia* are well known in several specimens visible in external and internal views. The skull roof is almost as long as broad forming in dorsal view a hexagon with edges of regular size, the anterior and posterior edges being slightly shorter than the lateral edges.

As in all coelacanths, the skull of *Rieppelia* is divided into two parts, namely the parietonasal shield and the postparietal shield, which are generally delimited by an intracranial joint. All the bones making up the skull roof – except the snout bones – are tightly sutured to one another. Parietonasal and postparietal shields are always preserved in close contact, even if the rest of the skeleton is lacking (e.g. PIMUZ T 5903) or was disturbed postmortem (e.g. PIMUZ T 1321). The two shields appear to be always tightly attached one another (Figs 2, S1a-b, S2), displaying a gap at the level of the supraorbitals and supratemporals (Fig. S3a-b), but without gap between the posterior parietals and the postparietals. When a gap is visible for taphonomic reasons the postparietals show areas of overlaps for suturing with the posterior parietals **(o.Pa) (**Fig. S3c**)**. Moreover, in ventral view the surface of the posterior parietals appears to be reduced in dorsal view, which is consistent with the fact that the posterior parietals overlap the postparietals. The intracranial joint **(i.j)** appears to be almost straight with some few indentations in dorsal view (Figs 2, S3) while it is completely straight in ventral view (Fig. S1). Therefore both shields were strongly sutured together (character 1) and the intracranial joint was not functionnal. This derived character shared with *Foreyia*[1] is unique among coelacanths.

The skull roof of *Rieppelia* departs from the standard morphology of latimerioids coelacanths by its parietonasal shield being approximatively the same length than the postparietal shield (Figs 2, S1a-b, S2, S3a-b) (character 2), a feature commonly found in Palaeozoic coelacanths.

2.2.1 Parietonasal shield

Beside the unusual morphology of some dermal bones, the skull roof pattern of *Rieppelia* is characteristic of coelacanths (Figs 2, S2, S3).

The snout bones have matching contours (Fig. S1a-b) and are independent from one another (character 3) although they are somewhat attached to one another (Fig. S2).

Each premaxilla (**Pmx**) is rectangular and carries a well-developed dorsal lamina (character 5) (Figs S1a-b, S2). No teeth have been identified along the oral margin of the visible premaxillae but it is hard to assert if they were absent in life or just lost postmortem (character 4).

Posterior to the premaxillae are a pair of small rostral ossicles **(ros.oss)** (Fig. S2). Wedged between the premaxilla and the lateral rostral is a single small bone (Figs S1a-b, S2). Together with the premaxilla, lateral rostral and tectal, this bone forms the ventral margin of a large opening. It lies in a similar position than the rostral ossicle located anteroventrally to the anterior opening of the rostral organ in *Latimeria* (the median postrostral of Jarvik[2], fig. 210; the unlabelled bone of Forey[3], fig. 3.1). Therefore, this bone is identified as a rostral ossicle and the large opening as the anterior opening for the rostral organ **(a.ros)**, which is then contained within separated rostral ossicles (character 6).

Lateral to the premaxilla is the lateral rostrals (**L.r**) (Figs S1a-b, S2). Usually, the shape of the lateral rostral in actinistians is relatively constant[3] and is composed of an elongated narrow tubular portion and an anterior part more developed that contacts the tectals on its dorsal margin, and has a more or less developed ventral process. The shape of the lateral rostral of *Rieppelia*, however, is unique among actinistians because its height is greater than its length. The dorsal region of the lateral rostral is overdeveloped and separate the unique tectal from the supraorbital series (Figs S1a-b, S2), while normally the tectal-supraorbital series is uninterrupted in coelacanths. Based on this bone pattern, it is assumed that the lateral rostral had fused with the neighbouring tectal, which results in the hypertrophied aspect of the lateral rostral. The ventral process of the lateral rostral is poorly developed (Fig. S2), as for instance in *Rhabdoderma*[3]. In internal view, the anterior tubular portion displays a small excavation opening on the ventral margin of the bone, which may correspond to the mark of the anterior nostril **(nos.a)** (Fig. 3). In this area, the anterior tubular portion is curved (Fig. S2) reinforcing the hypothesis that the

nostril opens at this location. At the posterodorsal margin of the tubular portion and at the junction of the preorbital and lachrymojugal is an opening interpreted as the posterior nostril **(nos.p)** (Figs S1a-b, S2).

In front of the anterior parietal and wedged between the nasal and the lateral rostral is a single rectangular tectal (**Te**). This bone is smaller than the nasal and as large as the rostral ossicles. It is identified as a tectal because it lies at a level anterior to the parietal and is adjacent to the triple junction of the sensory canal (Fig. S1a-b) as in *Latimeria*[3]. As mentioned above, this unique tectal is separated from the supraorbital series by the lateral rostral. It is supposed that the tectal that usually connects other tectals to the supraorbital series in coelacanths has been either lost or, more likely, incorporated into the dorsal portion of the lateral rostral. Indeed, the tectals seem to fuse with the neighbouring bones, namely the nasal or lateral rostral (Fig. S2).

The orbit is roofed by three large supraorbitals **(So)**. Including the unique tectal, the lateral series is then composed of four elements (character 12). The supraorbitals are as wide as the parietal (Figs 2, S2, S3a-b), like in *Foreyia*[1] and *Ticinepomis*[4] unlike the Latimeriinae. The supraorbitals are strongly sutured to one another and also with the parietals. This feature can be observed in internal view, where the first supraorbital overlaps the second supraorbital. The first anterior supraorbital flanks laterally the anterior parietal and the posterior parietal up to its mid-length, while the second and third posterior supraorbitals are only in contact with the posterior parietal. The third supraorbital has only a reduced contact with the posterior parietal and extend posteriorly behind the posterior level of the posterior parietal. It is worth noting that the third posterior supraorbital directly contacts (but does not suture with) the supratemporal, a condition unique among actinistians. Indeed, in all other actinistians the posterior most supraorbital is separated from the supratemporal by either the postorbital (e.g. *Laugia*[3]), a posterolateral process of the posterior parietal (e.g. *Latimeria*[3]) or the postparietal (e.g. *Whiteia*[3]). *Miguashaia* is the only other actinistians that has a condition different from the usual condition observed in actinistians. In it, the posterior most supraorbital directly contacts the intertemporal which is, as the supratemporal, a bone of the lateral series of the postparietal shield. In *Rieppelia*, the anterior supraorbital is the longest bones of the lateral series and the second supraorbital is as long as, or slightly longer than the posterior most supraorbital. The anterior and posterior supraorbitals are rectangular, while the second supraorbital is squarish. The third posterior supraorbital expends ventrally in such a way that its anteroventral margin participates to the posterodorsal coutour of the orbit. When regarded in anterior view, all supraorbitals are strongly angled in their middle, forming a sharp angle between the skull roof and cheek. This angle marks a strong crest running throughout the middle of the supraorbitals

series and crossing longitudinally the upper part of the opercle (Fig. S2a). In internal view, the posterior third supraorbital presents in the center of its posterior margin a ridged surface that corresponds to the insertion point for the opercular ligament **(op.lig)** (Figs 3, S1a-b). Along the internal margin forming the orbit, all three supraorbitals are reinforced with a thickening of the ossification marked with curved grooves parallel to the orbital margin (Figs 3, S1a-b). On the posterior third supraorbital, this thick bony portion is more reduced than on the other two supraorbitals. This reinforced structure is also present on the postorbital-squamosal bone and the preorbital, but is absent from the lachrymojugal. The function of this structure is not clear but it may correspond to a reinforcement of the orbital region. Sclerotic ossicles have not been observed in any available specimens, and it is then assumed that a sclerotic ring is absent (character 56).

The preorbital (**Preo**) (character 13) is round to ovoid in shape, and is sutured with the anterior supraorbital, the lachrymojugal and the lateral rostral (Figs 3, S1a-b, S2). In internal view, the orbital margin of the preorbital presents the same overdeveloped bony structure than observed on the supraorbitals (Figs 3, S1a-b). The anteroventral edge of the preorbital is notched for the exit of the posterior nostril (**nos.p**). Therefore, this notch should not be regarded as the corresponding notch observed on the mid-length of the ventral margin of the preorbital of *Foreyia* interpreted as the posterior opening of the rostral organ[3]. Consequently, the preorbital of *Rieppelia* is not pierced or notched for the posterior openings of the rostral organ unlike all other actinistians having a preorbital. As the cheek is well ossified and completely known, the posterior openings of the rostral organ **(p.ros)** supposedly opened in the anterior part of the orbital space (characters 46 and 47), in a position similar to that of *Latimeria*. It is excluded that the pore opening on the lachrymojugal (Figs 2, S3a-b) is the exit of the posterior opening of the rostral organ because in a large specimen (PIMUZ T 4985), this opening is an elongated pore running from the center of the bone to its posterior margin, a position incompatible with interpreting this pore as posterior opening of the rostral organ. Furthermore, after reconstruction, it seems that the parasphenoid is placed above this opening, thus excluding the identification of this pore as the posterior openings of the rostral organ, which should be located dorsal to this bone for anatomical reason.

Posteriorly to the rostral ossicles and tectals lies a pair of small squarish to rectangular nasals (**Na**) (Figs 2, S1a-b, S2). The nasals are slightly larger than the rostral ossicles but are considerably smaller than the parietals. The pair of nasals is sometimes separated by an internasal (**Ina**), strongly sutured with the neighbouring nasal (Fig. S2). The presence of this bone, however is not constant among the available specimens (Fig. S1a-b) (character 7).

Rieppelia has two pairs of parietals (**Pa**) (character 8). The parietals are square to rectangular, and they are approximatively of the same length (character 9), but the total surface of the anterior parietal is smaller than the one of the posterior parietal. The two pairs of parietals are then of similar size, as in *Ticinepomis*[4] and *Foreyia*[1], but unlike in the Latimeriinae. The sutural pattern between the anterior and posterior parietals is straight (Figs 2, S3a-b), resembling to the intracranial joint condition, or very slightly interdigitated (Fig. S2). In internal view, the anterior parietals appear longer than the posterior parietals (Fig. S2) unlike in external view where they are of the same length (Figs 2, S2, S3a-b). It means that the anterior parietal is overlapped by the posterior parietal. Therefore, although the sutural pattern between both pair is not interdigitated and complex as usually observed in actinistians, as for instance *Macropoma*[3], both pairs are however well fixed to one another. No raised areas are present on the parietals or the postparietals (character 10). A descending process (**v.pr.Pa**) is present on the posterior parietal (character 11) (Fig. S1a-b).

2.2.2 Postparietal shield

The postparietals (**Pp**) of *Rieppelia* are almost rectangular, being slightly longer than wide (Figs 2, S1a-b, S2, S3a-b). Thus, the postparietal is, as in *Allenypterus*, relatively equidimensional, which is considered by Forey[3] as an unusual feature in coelacanths. The lateral border is slightly curved in its posterior half in order to receive the supratemporal bone. Usually in coelacanths, the postparietal contacts the postorbital. However, in *Rieppelia*, the postparietal is not in contact with the postorbital-squamosal bone because it is separated by the posterior most supraorbital. This characteristic is possibly shared with *Foreyia*[1] in which all bones of the postparietal shield are fused together, and it is only observed in some few Palaeozoic actinistians, such as *Miguashaia*, *Gavinia* and potentially *Coelacanthus granulatus*. The postparietal bears a well-developed descending process **(v.pr.Pp)** (character 15) (Fig. 3). Apart of the supratemporal, there is no other canal-bearing bone, namely an intertemporal, along the postparietal (character 14).

Each postparietal is flanked by a smaller rectangular to ovoidal supratemporal (**Stt**) (Figs 2, S1a-b, S2, S3a-b). The supratemporal is in direct contact - but is not sutured - with the posterior third supraorbital, being separated by the intracranial joint. Therefore, the anterior margin of the supratemporal is not separated from the parietonasal shield by the postparietal bone, which is a condition unique among actinistians. The posterior margin of the supratemporal is rounded, fitting the concave anterior margin of the lateral extrascapular. The supratemporal bears a well-developed long and thin descending process **(v.pr.Stt)** (character 16) (Fig. 3).

The posterior margin of the supratemporal and the postparietal are at the same level resulting in a straight posterior margin of the skull roof (character 17) (Figs 2, S1a-b, S2, S3a-b), a characteristic commonly observed in Palaeozoic coelacanths such as *Allenypterus*[3], *Diplocercides*[5] or *Hadronector*[3].

The extrascapular series is represented by three large bones, one median (**Ext.m**) and a lateral (**Ext.l**) one on each side (characters 20 and 21) (Figs 2, S1a-b, S2, S3a-b). The lateral extrascapulars are almost square and equidimensional while the median extrascapular is slightly less wide, being then more rectangular to ovoidal. This configuration of three more or less large extrascapulars lying posteriorly to the supratemporals and postparietals is reminiscent of Palaeozoic coelacanths, for instance *Miguashaia*[6], *Diplocercides*[5], *Allenypterus* and *Hadronector*[3]. The three extrascapulars of *Rieppelia* gen. nov. are long and almost as wide as the postparietals, which is a remarkable feature. The extrascapular series occupies half the area of the postparietal shield. Extrascapulars hypertrophied to such a degree is unique among coelacanths, only possibly shared with *Foreyia* in which all the postparietal shield bones are fused together and form a hypertrophied dome[1]. The extrascapular series is strongly sutured to the skull roof (character 18). Indeed, the contact between the extrascapular series and postparietals-supratemporals develops as a long zig-zag line. Furthermore, when the bones are slightly disarticulated from each other, the posterior margin of the postparietals show narrow overlap areas (Fig. 2a-b) implying that the lateral and median extrascapulars are overlapping the postparietals. This situation indicates that the postparietals are firmly sutured to the extrascapular series. The extrascapulars are not flat bones but are bulging in their center in such a way that they form a small dome that recall somewhat the massive dome forming the head of *Foreyia*. The preservation of the available specimens of *Rieppelia* is not sufficient to state if the extrascapulars have been incorporated into the skull roof proper (character 19), as for instance in *Mawsonia* or *Axelrodichthys*[3].

2.3 Dermal bones of the cheek

The cheek of *Rieppelia* is unusual among actinistians in several respects. It is composed of an equidimensional lachrymojugal as large as the preopercle, a postorbital-squamosal compound bone and a very large opercle. The cheek bones (Figs 2a-b, 3, S3a-b) show areas of mutual overlap and are tightly sutured to each another (character 29), which is a unique condition among post-Palaeozoic coelacanths. The orbital space is small and was probably occupied entirely by the eye (character 55). The subopercle, the spiracular, a quadratojugal and the jugal (character 35) are clearly absent.

One of the most intriguing features of *Rieppelia* is a large bone lying in place of the usual postorbital and squamosal. This large bone is interpreted as the fusion of the postorbital and the squamosal **(Po+Sq)** (Figs 2, 3, S1a-b, S3a-b). It is unlikely that this bone is a squamosal alone because it bears the infraorbital sensory canal. The fusion between these two bones is unique among coelacanths. Nevertheless, some individuals of few taxa show evidences of fusion between other cheek bones, namely the squamosal and the preopercle. In a specimen of *Hadronector*, the squamosal and the preopercle have fused in a single and large bone ([3], fig.4.7a). A similar fusion between the squamosal and the preopercle was also observed in one specimen of *Latimeria* ([3], fig.4.2D). Contrary to *Hadronector* and *Latimeria,* in which this fusion corresponds to individual variations, the fusion between the postorbital and the squamosal in *Rieppelia* is a generic characteristic because it is observed in all the available specimens, from the juvenile to the adult stage. Another hypothesis would be that this bone represents rather a single large postorbital implying then that the squamosal has been lost. It is impossible to state weather the postorbital and squamosal have fused together or that the squamosal has been lost. However, as the cheek bones tend to fused together during phylogeny or ontogeny, as exemplified by *Hadronector* and *Latimeria*, we favour the hypothesis of a fusion.

The cheek of *Foreyia* is also peculiar. Because the large triangular bone previously identified as a lachrymojugal fused with a squamosal by Cavin et al.[1] is reinterpreted here as single lachrymojugal, the condition of the squamosal needs to be reassessed. The posterior cheek area is occupied by a small bone contacting the lachrymojugal but separated from the postorbital by a gap and a tiny bone placed below the opercle. Cavin et al.[1] identified with caution those bones as the preopercle and the subopercle. Assuming that the jugal sensory canal, which is enclosed in the squamosal, departs from the infraorbital sensory canal at the junction of the postorbital and lachrymojugal, as usually in coelacanths, the squamosal may be located in this area. Based on this statements, three hypotheses can be formulated: (1) the bone previously identified as the preopercle and the subopercle are the squamosal and preopercle, respectively; (2) the squamosal has been lost and is virtually located in the gap ventral to the postorbital; and (3) the preopercle is fused with the squamosal. As *Foreyia* is closely related to *Rieppelia*, a parsimonious approach implies that the second and the third hypotheses would be the most likely. The last one is the one preferred here because Cavin et al.[1] identified on this bone a notch for the sensory canal, which could in fact be a residual suture.

In summary, the structure of the cheek of *Rieppelia* and *Foreyia* is weird among coelacanths. Due to the peculiar condition of the postorbital-squamosal bone of *Rieppelia*, some characters

related to the postorbital (character 33) and to the squamosal (characters 36 and 37), useful for phylogenetic analyses, cannot be evaluated. Nevertheless, some other characters can be scored with confidence. The postorbital-squamosal bone is directly sutured to the posterior third supraorbital and presents no anterodorsal excavation (character 31). There is also no anterior process developing above the lachrymojugal (character 32). Regarding its position, the postorbital-squamosal lies behind the level of the intracranial joint (character 34). The posterior margin of the postorbital-squamosal is straight and abut against the opercle. The disposition of postorbital-squamosal, posterior supraorbital and opercle implies that there is no available space for a spiracular, which is then regarded as absent (character 30). Indeed, there is no available space for this bone anterodorsally above the opercle, and it has never been observed.

The lachrymojugal **(L.j)** of *Rieppelia* is bone with a very peculiar shape (Figs 2, 3, S3a-b). The bone is almost equidimensional and its length is only slightly greater than its width, giving to the lachrymojugal an appearance of a relatively short and thick bone with a roughly triangular shape (character 43). This shape is reminiscent of the lachrymojugal of *Foreyia* (the lachrymojugal+squamosal in[1]) and, to a lesser extent, of *Ticinepomis*[4]. The lachrymojugal of *Rieppelia* contrast then with the usual condition observed in coelacanths that have a more or less elongated and curved lachrymojugal. Due to the very strong deformation of the lachrymojugal, it is impossible to state if the anterior end is angled and/or expanded (character 42). Anteriorly, the lachrymojugal lies in contact with the preorbital (character 44) and the lateral rostral. At the junction of the preorbital and lateral rostral, the anterodorsal margin of lachrymojugal is notched, presenting a cupular bony surface anteriorly directed (PIMUZ T 4985), which accommodates the exit of the posterior nostril. Therefore, the lachrymojugal is marked by the posterior nostril (character 45) but in a lesser extend as for instance in *Latimeria*[3].

The preopercle **(Pop)** is a large bone (character 38) lying below the postorbital-squamosal and the lachrymojugal (character 40) (Figs 2, 3, S3a-b). The bone has an undifferentiated shape (character 39) and is roughly croissant-shaped being very similar to the preopercle of *Whietia woodwardi* ([3], figs 4.14 and 4.15). The dorsal margin of the lachrymojugal presents large surface of overlap **(o.s)** (Figs 2a-b, S3a-b) for the postorbital-squamosal bone and the lachrymojugal. Therefore, all bones of the cheek are strongly sutured together. The preopercle and the opercle lies in direct contact (Fig. 2a-b) in such a way that there is no space left for the presence of a subopercle, which has never been observed in the available specimens, and consequently regarded as absent (character 41). Moreover, as the opercle of *Rieppelia* covers the area normally occupied by the opercle and subopercle in other coelacanths, a fusion of these two bones cannot be excluded during ontogenesis in the genus. Interestingly, the preopercle of

the juvenile/newborn specimen (Fig. 3), which is visible in internal aspect, present a strong mark/groove in its first anterior third. The latter may be interpreted as a suture between two bones, a feature that has never been observed in external view on available specimens. As this feature is observed in a specimen regarded as a juvenile/newborn specimen, this suture may correspond to a remnant suture of an earlier ontogenic stage. Before the synthetic work of Forey[3], the preopercle of actinistians was often named preoperculo-quadratojugal[5] because of the presence in some taxa of a pit line considered to be homologous to the quadratojugal pit line found in other sarcopterygians and actinopterygians. Forey[3] calls this bone preopercle because he never found any evidence of a fusion (i.e. a true fusion observed by the mean of two centers of ossification) between the preopercle and quadratojugal, and because the presence of the preopercular canal is regarded as more important than the pit line. On the preopercle of *Rieppelia*, there is only one center of ossification located in the first posterior third of the bone and radiating on the entire bone. Nevertheless, it is difficult to interpret this mark in the preopercle of the juvenile/newborn specimen as something else (i.e. sensory canal) than a suture. The case of *Rieppelia* is thus a possible clue of a fusion of the preopercle with the quadratojugal that occur during evolution, and still visible at early ontogenic stage, especially for those actinistians with a croissant-shaped preopercle.

The opercle **(Op)** is a very characteristic large bone, as long as deep (Figs 2, S1a-b, S3a-b, S4). The opercle, ovoid-to-triangular shaped, is hypertrophied, covering a greater area than the whole surface formed by the cheek and supraorbitals together. The anterior margin contacting the cheek is straight (Figs 2, S4) or slightly concave (Fig. S3a-b). The margin contacting the postparietal shield is convex. From its posterodorsal corner to the ventral corner, the posterior margin is straight (Fig. S3a-b) to sinusoidal (Fig. S4).

The antero-dorsal corner of the opercle produced as a small ridged process (Fig. S4) which faces a similar ridged surface on the posterior-most supraorbital (Figs 3, S1), providing points of attachment for the opercular ligament (**op.lig**). In coelacanths, the articulation between the opercle and the skull is known in some taxa as *Coelacanthus granulatus*, *Laugia*, *Libys polypterus*, *Macropoma* and *Latimeria*[3]. In those taxa, the opercle is linked to the skull by a ligament that attached on a ridged surface located on the lateral margin of the supratemporal. Therefore, this point of attachment is different from the one of *Rieppelia.* The opercle is not a flat bone and is strongly angled dorsoventrally along an axis connecting the articular facet to the posterodorsal corner of the bone. This angle is marked by a stronger ornamentation forming thus a strong crest that is the continuation of the crest observed on supraorbitals.

2.4 Lower jaw

The bones of the lower jaw of *Rieppelia* are never preserved articulated among the available specimens (Figs S1a-b, S2), except to some extent, in the holotype (Fig. 2a-b) and the juvenile/newborn specimen (Fig. 3). When reconstructed (Figs 2c, S8), the lower jaw is small but with an organization comparable to that of other coelacanths.

The articular facets have not been observed on any specimens and it is then not possible to assess if the retroarticular and articular are co-ossified or not (character 57).

The angular **(Ang)** is the largest bone of the mandible (Figs 2, 3, S1a-b). The angular is parallel-sided in the same way as in *Ticinepomis* spp.[4,7,8]. Its ventral margin is very slightly concave. The deepest point is located approximately midway along the length of the angular.

The prearticular **(Part)** is a large bone, which has its mesial surface covered by round to ovoid sockets that accommodated teeth (Fig. S1a-b).

In the juvenile/newborn specimen only, the splenial **(Spl)** and the dentary are observed in articulation (Fig. 3). The splenial appears to be deep with parallel margin and is as long as the dentary as in *Ticinepomis* spp.[4,7,8] and *Foreyia*[1]. The ventral margin of the splenial is very slightly concave but together with the angular, the ventral margin of the lower jaw appears to be very curved. The anteroventral portion of the splenial is slightly curved downward, similarly, in the same way, but however less than in *Ticinepomis* spp.[4,7,8], *Foreyia*[1], *Dobrogeria*[9] and *Whiteia woodwardi* ([3], figs 5.9a and 5.9c).

The dentary **(De)** is only observed in the juvenile/newborn specimen (Fig. 3). It forms a long process with a straight oral border extending posteriorly as in *Ticinepomis* spp.[4,8] and *Foreyia*[1]. In its mid part, the dentary appears to be as wide as the splenial and becomes slightly narrower anteriorly. The posterior margin of the dentary forms a small hook but unfortunately the exact relationship between the dentary and the angular is not clear, preventing confirmation that the dentary is hook-shaped (character 58). It is also impossible to determine the condition of the lateral swelling on the dentary (character 59) but this feature is probably absent when the general shape of the mandible is considered.

The dentition of *Rieppelia* is very distinctive among coelacanths. In the holotype (Figs 2a-b, S5a) and juvenile/newborn specimens (Fig. 3), rows of teeth are observed directly above the oral margin of the bones of the lower jaw. All teeth are curved and measure between 1 mm to 1.3 mm long and are found separate from the dentary (character 60). Although all teeth are of the same size, few are gathered by clusters of three curved teeth forming small toothed elements, whereas the majority lies in pack but appears to be free, independent to each other. It is excluded

that these teeth belong to the basibranchial apparatus as they are always observed in the oral area of the jaws in many specimens (Figs 2a-b, 3, S1, S2, S3a-b).

The free teeth are considered to be shedded teeth of the dentary tooth plates **(t.p.d)** that were borne by the dentary (Fig. 2a-b). They probably laid free in the skin as in *Latimeria*, which is considered to be a derived condition[3]. A total of about 200 single teeth have been counted in the holotype of *Rieppelia* (PIMUZ T 5902). Above the left angular is a bony element, interpreted as a tooth plate, on which 22 teeth are packed. Regarding the total number of single isolated teeth, it would mean that the dentary was overlain by at least 8 distinct tooth plates. In *Latimeria*, 8 and 14 small dentary tooth plates are present in an embryo and an adult, respectively, each one supporting a shagreen of tiny teeth[3,10]. *Coelacanthus granulatus* has six tooth plates with 40-50 acutely conical teeth[3].

Clusters of three teeth are considered to represent coronoids **(Co)** (Figs 2, 3, S1, S2, 3a-b) and few to dermopalatines. The bony base on which the teeth are born is curved ventrally (Fig. S1c). Those clusters of three teeth have a very characteristic shape, resembling the tricuspid parasymphysal tooth whorls present in Onychodontiformes, such as *Onychodus*[11], although proportionally much smaller. Although their resemblance, these three-toothed elements in *Rieppelia* are not identified as parasymphysal tooth whorls as in *Onychodus*. Indeed, the holotype (Fig. 2a-b) and juvenile/newborn specimens (Fig. 3), these elements are found along the oral margin of the mandible. Four clusters of three teeth (for a total of six on the fossil) are observed on the right lower jaw of the juvenile/newborn specimen (Fig. 3) and nine to eleven clusters are identified in the holotype (Fig. 2a-b). Based on this situation, and especially in the juvenile/newborn specimen (PIMUZ T 3376), we assume that there are four coronoids on each hemi-mandible (character 62) and that the situation is then similar to that of all other coelacanths – with the only exception of *Diplocercides* that has three coronoids[3]. Furthermore, we interpret the additional clusters of three teeth observed in the holotype (PIMUZ T 5902) as dermopalatines that are then structurally not different from the coronoids. A similar case occurs in *Ticinepomis peyeri* that has coronoids and dermopalatines sharing the same structure and shape of teeth[4]. As there is no size variation between the coronoids, the coronoid opposite to the posterior end of the dentary is considered to be not modified (character 63). The teeth are pointed and smooth (character 65), and are identified as fangs (character 64). The coronoid structure of *Rieppelia* is thus different from the one of *Latimeria* that is composed of a dental plate on which rests a large fang, one mid-sized caniniform tooth and numerous small round-shaped teeth[12].

In the juvenile/newborn specimen, a fragmented and ill-defined bone located above the right angular is interpreted with caution as the principal coronoid **(p.Co)** (Fig. 3). This bone has shifted from its original position meaning that it was separated from the angular (character 61).

Only one clearly identifiable gular plate **(Gu)** visible in external view has been observed (Fig. S5b). The lateral border is curved while the medial border is approximately straight. The anterior border is rounded and wider than the posterior margin. Posteriorly, the bone tapers to a sharp point. From the posterior tip to the middle of the bone, a bulge runs along the anteroposterior axis parallel to the lateral edge. This bulge may possibly represent the mark of an internal ridge. In *Latimeria* and *Megalocoelacanthus* this ridge is related to the insertion point of the anterior and posterior ramus of intermandibular muscle[13]. In the juvenile/newborn specimen, there is a proportionally large ossification that we interpret with caution as a possible gular bone (Fig. 3). Its posterior portion forms a pointed tip. The lateral and mesial margins are parallel and the anterior margin is straight, giving to the bone an almost rectangular shape with a pointed posterior portion. Similarly to the other identified gular bone, there is a strong crest running from the middle of the bone in the direction the posterior.

2.5 Sensory canals, cranial nerves and pit lines

In *Rieppelia*, the strong ornamentation of its dermal bones makes difficult to detect the opening of the sensory canals but a careful observation indicates that they are present on the bones of the skull roof, the cheek and the lower jaw. The specimens preserved in internal view display grooves on the internal surface of the bones that are interpreted, depending of their location, as the path of the cranial nerves or as the sensory canals. These structures may also be observed in volume but representing the filling of the underlying canals that have been removed (i.e. for instance part and counterpart). In some part, especially in the snout, these structures seem to represent the sensory canal together with the path of the cranial nerves, which have been compressed together during fossilization. Indeed, regarding the disposition of the sensory pores and the observed grooves, it appears that, in the snout area, the sensory canals may have been located just above the nerves. Such a situation would not be surprising as in the living *Latimeria* the nerves and the sensory canals are located one above the other or close to each other[10,14]. In the middle part of the skull roof, namely on the parietals and postparietals, the sensory canals are placed laterally to the path of the groove, here representing the path of the nerve. In this part the nerve sends branches to innervate the supraorbital sensory canal. The situation of the nerves in the posterior part, the cheek and the lower jaw remains unknown.

2.5.1 Sensory canals

Generally, the pores for the sensory canals are hard to detect because of the strong ornamentation of the dermal bones and especially because it is difficult to distinguish a true pore from the sockets of lost a lost ornament. On the skull roof, the pores of the sensory canal are numerous and small on the snout and become rarer and smaller posteriorly, being absent on the extrascapular series (Fig. S2). Therefore, the ethmosphenoid portion of *Rieppelia* must have been a more sensitive region than the otico-occipital portion.

On the snout, most pores of the supraorbital sensory canal **(p.so.s.c)** opens at the sutural contact between ossifications, but some pores are located within the bones, namely on the tectal and the premaxillae (Fig. S2). The exact location of the ethmoidal commissure is difficult to detect. However, the positioning of the pores (Fig. S2) and the path of the underlying nerve, which merges with the supraorbital canal (Fig. S5c), indicate that it was running between the rostral ossicles as, for instance, in *Latimeria*[3]. Unlike in the latter where it lies free in the skin[3], the ethmoidal commissure of *Rieppelia* appears to be enclosed within the dermal bones. The exact course of the supraorbital sensory canal **(so.s.c)** can be directly observed in one specimen (Fig. S2a-c) in which an ancient preparation has removed the external bony surface forming the roof of the canals, thus revealing them. This specimen indicates that the path of the sensory canal follows the sutural course between the parietals and the supraorbital series (character 22) but can diverge slightly from the sutural contact within the posterior parietal. The canal opens through few small pores along the sutural contact between the supraorbital series and the parietals (Fig. S2). Regarding the general situation on the skull roof, the openings of the supraorbital sensory canal should be considered as opening at the sutural contact between bones (character 23). The floor of the supraorbital sensory canal is pierced by eight small foramens (Fig. S3a-b,d). The fossilization shows clearly that those foramens are medially directed and that they correspond to foramens for branches of the superficial ophthalmic branch of the anterodorsal lateral nerve **(f.br.s.opth)** (Fig. S3d). At the level of the anterior parietal, two to three anterior foramens are located very close to each other. Posteriorly, the foramens are further apart and almost regularly spaced. The supraorbital sensory canal of *Latimeria* is innervated by seven regularly spaced ramules sent by the superficial ophthalmic ramus of the anterodorsal lateral line nerve ([15], fig. 21). Therefore, compared to *Latimeria*, it seems that *Rieppelia* has one more neuromast innervating the supraorbital canal.

There are few openings for the otic sensory canal **(p.ot.s.c)** (Fig. S3a-b). At the margin of the supratemporal and the postparietal, there is a small pore formed by two neighbouring notches.

Few round pores are located in the middle of the supratemporal indicating that the otic sensory canal runs through the center of the bone, as in other coelacanths. At the posterior margin of the supratemporal is a notch (Fig. S3a-b) or a pore (Fig. S2). On the surface of the postparietal, there is no pore for the median branch of the otic canal (character 24) and no anterior branches of supratemporal commissure (character 25).

The extrascapular series is devoid of any pore. However, the supratemporal commissure **(stt.com)** clearly runs through the posterior quarter of the extrascapular series as indicated in one specimen (PIMUZ T 1755). The triple junction of the otic, supratemporal and lateral line sensory canals is neither observed externally nor internally. However, because of the shape of the skull roof, it is clear that the triple junction occurs in the lateral extrascapular.

At the suture between the middle and posterior supraorbital opens a small to medium-sized ovoid pore **(p.io.s.c)** indicating that the canal turns towards the cheek to extend as the infraorbital sensory canal (Figs S2, S3a-b). No openings for the infraorbital sensory canal are observed on the postorbital-squamosal bone. Therefore, the exact path and development of the infraorbital sensory canal within this compound bone is unclear (characters 48 and 49). The lachrymojugal is perforated in its middle by a large rounded pore (character 52) (Figs 2, S3a-b), which is in some specimens surrounded ventrally by much smaller pores, forming a line perpendicular to the ventral margin of the bone (Fig. S3a-b). The large pore indicates the path of the main infraorbital sensory canal and the small pores represent short secondary branches. In a large specimen (PIMUZ T 4985), this large pore appears as an elongated pore running from the center of the bone to its posterior margin. Apart the pore(s) observed on the lachrymojugal, the other bones of the cheek show no visible pores. Therefore, the exact pattern of the cheek sensory canals (characters 50 and 51) and the junction between the infraorbital and the jugal sensory canals are unclear. Interestingly, the situation observed on *Rieppelia* is the opposite of that observed on *Foreyia*, which has no pore on its lachrymojugal (lachrymojugal+squamosal of [1]) but has pores on the postorbital[1].

Regarding the juvenile/newborn specimen (Fig. 3), the third posterior supraorbital, the postorbital-squamosal bone and the preopercle are marked on their internal side by shallow grooves that could correspond to the sensory canals. The groove marks the postorbital-squamosal bone from the dorsal margin, near its mid-length, then runs to the center of the bone, where it diverges into two grooves. One groove runs lateral to the thick border of the ossification around the orbit in direction of the lachrymojugal. The second groove runs posteriorly from the latter groove towards the preopercle. The anterior groove may correspond to the infraorbital sensory canal **(io.s.c?)** while the posterior one to the jugal sensory canal **(j.s.c?)**. This

interpretation would indicate that the triple junction of the infraorbital and jugal sensory canals is located in the middle of the postorbital-squamosal bone, which is odd for coelacanths. The posterior groove leaves the postorbital-squamosal bone and enters the preopercle at its posterior dorsal border. In the center of the preopercle, the groove loops anteriorly in direction of the mid ventral margin of the bones, where it presumably continues in the lower jaw. Topologically, this groove may correspond the preopercular sensory canal **(pop.s.c?)**. Another interpretation would be that the anterior groove on the preopercle, currently identified as a remnant suture, is the preopercular sensory canal. This configuration would make the triple junction of the sensory canals occurring at a more expected location for a coelacanth. However, this situation makes difficult to identify the posterior grooves on both the postorbital-squamosal bone and the preopercle. We do not retain this last hypothesis.

In the lower jaw, the mandibular sensory canal **(m.s.c)** runs along the mid-height of both the angular (Fig. S1a-b) and splenial (Fig. 3). There is no indication if the mandibular sensory canal forms posteriorly a subopercular branch (character 66). The canal opens to the surface of the angular (Fig. 2) along its mid-height through few and mostly irregularly spaced ovoid and round pores **(p.m.s.c)**. Regarding the two angulars preserved in the holotype (PIMUZ T 5902), the posterior pores are not placed at the same level on both bones, being located more dorsally on the right angular. The pores are then not all located along a single straight line and some are shifted from the line. In the juvenile/newborn specimen (Fig. 3), the mandibular sensory canal enters into the splenial and runs along the mid-height as indicated by a strong groove on the inner surface of the bone. The symphyseal margin of the splenial is notched such that it forms a large symphyseal pore upon contact with its antimere. This character is shared with a few other coelacanths, notably *Ticinepomis* spp.[4], *Foreyia*[1], *Whiteia woodwardi* (drawn but not labelled or described in [3], fig. 5.9A) and potentially *Luopingcoelacanthus* (not described but suggested by the illustrations provided by Wen et al. ([16] figs 1 and 2A). Due to the poor preservation of the dentary, the situation of the dentary pore cannot be assessed (character 67).

2.5.2 Otic lateral line nerve

In internal view the bones of the skull roof are crossed by strong grooves that we interpret has the path of the superficial ophthalmic ramus of the anterodorsal lateral line nerve **(s.opth)** (Figs S1a-b, S5c). This groove can be well observed especially in the specimen PIMUZ T 1638 (Fig. S1a-b). The groove starts to mark the bone anteriorly to the mid-length of the postparietal. It suggests that the sensory ganglion of the superficial ophthalmic ramus of the anterodorsal lateral line nerve was probably located in the otico-occipital portion as in *Latimeria*[14]. The

groove exits the postparietal to enter the posterior parietal by crossing the intracranial joint. The groove runs along the mesial side of the parietal descending process and passes across the middle of the center of ossification of the posterior parietal. In the anterior parietal, the groove runs between the lateral margin and the center of ossification of the bone. At midway, the groove appears to diverge medially to form a long groove that passes through the center of ossification. The exact end of this medial groove is not obvious but it gets very close from its antimere. In another specimen, namely PIMUZ T 1755 (Fig. S5c), this medial groove is absent either because of taphonomic reason or because the medial groove observed in PIMUZ T 1638 is not correctly interpreted and is rather a crack. However, the first hypothesis is preferred here because the walls of the medial groove present the same thin bony surface observed in the wall of the main groove, in contradiction with the second hypothesis. Therefore, compared with *Latimeria*[14,15], this groove may possibly correspond to the major ramule that innervates the sensory epithelium associated with the two posterior rostral tubes. The location of this groove at the level of the anterior portion of the orbital space also strengthens the hypothesis that the openings for the posterior rostral tubes are located in the anterior part of the orbit, just in front of the eye, as in *Latimeria*. In PIMUZ T 1755 (part and counterpart), we detected three laterally directed groove (better seen in 3D on the part) at the level of the anterior parietal (Fig. S5c), which seem to correspond to the three foramens observed on the floor of the supraorbital sensory canal of PIMUZ T 5903 (Fig. S3a-b,d). On PIMUZ T 1638, the foramens are difficult to detect and seem to correspond to enlargements of the groove in which is located a deepening directed towards the inside of the bone, i.e. towards the sensory canal (Fig. S1a-b). Comparing the localisation of the groove and the openings for the supraorbital sensory canal (Figs S1a-b, S2, S3a-b,d), it appears that the supraorbital sensory canal lies lateral to the groove at the level of the postparietals up to the anterior parietals. In the snout, however, the situation is different and the openings for the supraorbital sensory canal are located directly above or very close to the groove for the nerve. From this perspective both structures may have been merged together during fossilisation making hard to differentiate them. Thus, at the junction between the anterior parietal, the tectal and the lateral rostral, the groove splits into two grooves (Fig. S1a-b). One groove runs within the lateral rostral and ends lateral to the anterior opening of rostral organ. The other groove continues through the center of ossification of the tectal and the nasal. At the level of the nasal and rostral ossicles, the groove gets closer medially to its antimere and then finish by forming a loop laterally, which can be clearly observed in PIMUZ T 1755 (Fig. S5c).

2.5.3 Pit lines

Because of the strong ornamentation of the bones, the absence/presence of grooves for pit lines is difficult to assess, but a carful observation shows that none is present on the postparietals (characters 26 and 27) and on the cheek bones (character 53) of *Rieppelia.* It means that they must have lain superficially in the skin in life, if present. It is worth noting that on the angulars of the holotype (PIMUZ T 5902), there are a narrow groove crossing vertically the middle of the bones. This groove is continuous on the left angular while it is divided in two section on the right angular. Those structures are identified as a fracture produced by the curved shape of the bone rather than an oral pit line. However, as we are not sure about this identification, we prefer to let the conditions of the oral pit line as unknown in the phylogenetic analysis (characters 69 and 70). The preservation of the only clearly identified gular plate does not allow determining if it is marked or not by a pit-line.

2.6 Neurocranium and parasphenoid

The neurocranium and the parasphenoid of *Rieppelia* are only known from the juvenile/newborn specimen (PIMUZ T 3376). Therefore, some anatomical features (i.e. degree of ossification, size, relative position of bones) must be then regarded with caution because possibly constrained by the early ontogenic stage of the specimen. Moreover, the very flat conservation of the specimen prevents to score most of the braincase characters for the phylogenetic analysis (characters 74, 76, 83, 84, 85, 86, 87).

The neurocranium is divided into the ethmosphenoid and otico-occipital portions as usually in actinistians. The braincase is not preserved as a single element and the orbitosphenoid and basisphenoid regions are thus regarded as not co-ossified being separate from each other (character 71) (Fig. 3).

No vomers have been identified in the available specimens (character 81) but their true absence remain questioned here.

The basisphenoid **(Bsph)** has no basipterygoid process (character 73) (Fig. 3). The condition of the suprapterygoid processes cannot be assessed (character 80). The antotic processes **(ant.pr)** develops dorsally as an overlapping surface for the descending processes of the posterior parietals. The processus connectens **(pr.con)** lies above the ventral border of the basisphenoid. The antotic processes and the processus connectens are very short proportionally to the basisphenoid.

The parasphenoid **(Par)** is found apart from the basisphenoid (Fig. 3). Unfortunately, because the scattered preservation, it is impossible to know how was the contact between both bones (character 72). However, the position of the processus connectens may suggest that the parasphenoid was probably not meeting the processus connectens. The parasphenoid is a long bone that narrows in its mid-length and expands anteriorly where two parallel ridges marks as small lateral wings **(a.w.par)** (character 79). As the bone is strongly flattened due to taphonomic processes, it must have been narrower in life. There is no opening in the parasphenoid for the buccohypophysial canal, meaning that it was closed (character 78). Because the parasphenoid is preserved in dorsal view, its dentition cannot be observed (character 77).

The otico-occipital portion of the braincase is well developed and composed of a separate basioccipital and a pair of prootics (character 75) that surround two catazygal plates (Fig. 3). Therefore, the otico-occipital portion of *Rieppelia* presents the derived condition by being ossified as separate elements, unlike plesiomorphic coelacanths having this portion ossified in a single element, as for instance *Diplocercides*[3].

The basioccipital **(Boc)** forms the posterior limit of the basicranial fenestra (Fig. 3). Its posterior margin is partially covered by the cleithrum on the juvenile/newborn specimen (PIMUZ T 3376), but this margin can be indirectly observed. The exact nature of the suture between the basioccipital and the prootic (character 82) is hard to see because most of the sutural contact is hidden by the anocleithrum.

The pair of prootics **(Pro)** are relatively broad with an approximately triangular shape (Fig. 3). Their posterior portion, where they suture with the basioccipital, is broad in such a way that they do not form an elongated and thin posterior wing as for instance in *Holophagus* ([3], fig. 6.9).

An anterior and a posterior catazygals **(a.Cat; p.Cat)** plates occupy the basicranial fenestra (Fig. 3). Those two plates embraced ventrally the notochord in life. The posterior catazygal has a trapezoidal shape with an anterior margin slightly swollen, and the anterior catazygal is rectangular-shaped. The anterior catazygal is larger than the posterior catazygal, similarly as, for instance, in *Whiteia*[3].

2.7 Palatoquadrate, hyoid and gill arches

2.7.1 Palatoquadrate

The bones of the suspensorium are rarely preserved in available specimens. These bones seem to be all present in the juvenile/newborn specimen (PIMUZ T 3376) but their identification should be regarded with caution.

The pterygoid **(Pt)** is probably the best identified bone of the suspensorium. In the juvenile/newborn specimen (PIMUZ T 3376), this bone forms a low and elongated triangle (Fig. 3). Parallel to the posterior margin runs dorsoventrally a strong crest. Along the posterior margin of this crest the surface appears to develop in such a way to receive and contact the bones of the quadrate and/or of the metapterygoid. Anterior to the maximal height of the bone, its outline forms a triangular outline that tapers anteriorly. The ventral margin is slightly concave. On the ventral margin, at the level of the insertion of the quadrate is a small structure budging outward. It is unclear if it represents a small ventral swelling of the pterygoid (character 88) or an artefact of preparation/fossilization. A similarly triangular shaped bone is preserved in PIMUZ T 1755 (Fig. S5d). Its shape, however, present an anterior portion more elongated and thinner than in the juvenile/newborn specimen (PIMUZ T 3376), which is not surprising as this specimen is the largest known specimen of *Rieppelia*.

The quadrate **(Q)** is a long and rectangular bone (Fig. 3). The dorsal margin is flat. The lateral margins are parallel and curves slightly anteriorly. The ventral portion of the quadrate is formed by the double condyle for articulation with the lower jaw. It should be cautioned that in the juvenile/newborn specimen (PIMUZ T 3376), the double condyle has been slightly shifted during fossilisation. The general shape is reminiscent of the quadrate of *Diplurus newarki* ([17], fig. 6 and pl. 13.1).

The identification of the metapterygoid is uncertain in the juvenile/newborn specimen (PIMUZ T 3376) as bones of various shapes can be interpreted as such, and we leave the identification unresolved.

In the juvenile/newborn specimen (PIMUZ T 3376), a pair of rectangular bone are interpreted as ectopterygoids **(Ecpt)** (Fig. 3). One of them visible in mesial aspect presents some rounded teeth similar to that borne on the pterygoid.

Close to the pterygoid are two small triangular bones that are interpreted as the autopalatines **(Aut)** (Fig. 3). This identification is reinforced because one of them is in direct contact with one of the ectopterygoid.

2.7.2 Branchial arches and urohyal

In the juvenile/newborn specimen (PIMUZ T 3376), one ceratohyal and 8 to 9 ceratobranchials are preserved (Fig. 3). Therefore, there are at least four pairs of ceratobranchials **(Cb)**. One of the supposedly ceratobranchials **(Cb?)** have a shape recalling an epibranchials. Usually there are five pairs of ceratobranchials in actinistians[3]. Nevertheless, the situation of the branchial apparatus of *Rieppelia* is reminiscent of the one of *Dobrogeria*. In this latter, four pairs of broad ceratobranchials, although a fifth was probably present, and two small potential epibranchials are known[9].

The ceratohyal **(Ch)** has a small process expending from its central ventral border, giving to the bone the characteristic shape of the ceratohyal of coelacanths[3] (Fig. 3). The central position of this process on the ceratohyal is reminiscent to that of, for instance, *Ticinepomis* spp.[4], *Dobrogeria*[9] and *Luopingcoelacanthus*[16]. The situation is nevertheless different from other coelacanths as, for instance *Latimeria* and *Macropoma*, in which the process occurs at the two-thirds distance from the anterior end of the ceratohyal[3].

The urohyal **(Uhy)** is preserved in the holotype (PIMUZ T 5902) (Fig. 2a-b) and in other specimens (Figs 3, S5e-f). The posterior extremity presents a bifid tip with a V-shaped slit between both processes. Two ridges run from the tip of the posterior processes, converge toward the centre and diverge again anteriorly, giving to the bone a symmetrical shape. Unlike in the posterior portion, there is a thin layer of bone between the two anterior ridges. The anterior portion is slightly narrower than the posterior portion. In coelacanths, the shape of the urohyal is said to be very constant[3]. It develops as an elongated bone that is narrow anteriorly and expanded posteriorly with a bifid tip, exemplified by *Latimeria* and *Macropoma* ([3], figs 7.6B and 7.7). Therefore, the urohyal of *Rieppelia* departs from the usual condition in coelacanths by being short and ovoid shaped. In *Diplurus newarki*, Schaeffer ([17], pl. 6.4) identified a similarly shaped bone as a possible urohyal, an identification that is likely exact in the light of the urohyal of *Rieppelia*.

In the holotype (PIMUZ T 5902), there is large and diamond-shaped bone **(Bb?)** (Fig. 2a-b), which recalls the mostly cartilaginous basibranchial of *Latimeria* ([3], figs 7.6A-B). In the juvenile/newborn specimen (PIMUZ T 3376), some tiny round teeth that are born on an ovoid surface, which may represent one or more basibranchial tooth plates **(t.p.Bb?)** (Fig. 3). It is also possible that the ovoid surface represents directly the basibranchial bones but the situation is unclear due to the preservation and the high compression of the bones. Therefore, the conditions relative to the basibranchial tooth plate cannot be determined (character 89 and 90).

2.7.3 Hyoid arch

In some specimens of *Rieppelia* (PIMUZ T 31, 1639 and T1755) is a large and rounded bone, often poorly preserved (Fig. S5g). This bone is very thin and shows lines of growth parallel to its external margin. The shape of this bone recalls the hyomandibula of *Latimeria* ([10], pl. XLIII). In coelacanths, exemplified by *Latimeria*, the hyomandibula is cartilaginous but can be exceptionally partly ossified as in *Laugia*[3]. Therefore, the bone observed in *Rieppelia* may potentially represent an ossified hyoidmandibula.

An hourglass shaped bone with slightly rounded extremities is interpreted as the symplectic (Fig. S5g-h). The bone is reminiscent to the symplectic of other coelacanth as for instance *Latimeria* ([10], pl. XLL; [3], fig. 7.4). In coelacanths, the shape of the bone is also relatively constant and variates only in size.

2.8 Postcranial skeleton

The postcranial skeleton, comprising the axial skeleton, the basal support of the second dorsal fin and some bones of girdles and fins are preserved articulated in the holotype (PIMUZ T 5902) (Fig. 2a-b) and in some other specimens (PIMUZ T 3376 and 4985) (Fig. 3). It should be warned that due to the early ontogenic stage of the juvenile/newborn (PIMUZ T 3376) individual the girdles, except the pectoral girdle, were not ossified, which explain why they are not observed in this specimen. Furthermore, the small size of the specimen makes it difficult to distinguish the different bones of the axial skeleton and of the fin rays.

Of the available specimens, none show ossified lung plates, meaning that an ossified lung was likely absent in life (character 97).

2.8.1 Axial skeleton

The axial column of *Rieppelia* is very short with a total of 35 neural arches **(n.a)** (character 93) as counted on the holotype (PIMUZ T 5902) (Fig. 2). A similar maximal count is obtained for the neural arches in the juvenile/newborn specimen (PIMUZ T 3376). This low number of neural arches is one of the lowest known among coelacanths, identical to that of *Foreyia*[1]. This low number of neural arches is not related to the body size because *Rieppelia* is a coelacanth twice as large as *Foreyia*. The proximal most neural arches are badly preserved in the holotype (PIMUZ T 5092) and are covered by the opercle, making impossible to distinguish if they are broader than the other neural arches (character 95). The dorsal extremity of the abdominal neural arches is thin while the caudal neural arches have an enlarged distal extremity for the contact with the radials.

The haemal spines **(h.a)** are poorly preserved and are consequently quite difficult to observe in the holotype (PIMUZ T 5902) because they are tightly compressed on top of each other (Fig. 2a-b). Nevertheless, there is an indication of about 14-16 haemal rods. The posterior neural arches, and likely the haemal arches, are spaced and not abutting (character 94).

No long ossified ribs have been identified in any available specimens (character 96).

A count of 13 to 16 dorsal and 11 to 14 ventral radials **(Ra)** have been done in the holotype (PIMUZ T 5902) (Fig. 2a-b). The rods are symmetrical, flatten and large with proximal and distal expanded extremities. The first anterior dorsal and the two or three anterior ventral radials seem to support no fin rays.

2.8.2 Unpaired fins

The unpaired fins of *Rieppelia* include two dorsal fins, one anal fin and the caudal fin with a supplementary lobe (Fig. 2). The rays of all fins, unpaired and paired together, are hollow as in other coelacanths, and are unbranched (character 107). The rays are unsegmented proximally and their distal portion is segmented. There is a gradual transition from the unsegmented to the segmented portion. Denticles (Fig. S7f) are present on the fin rays of all fins. It is worth noting that on the juvenile/newborn specimen (PIMUZ T 3376), the denticles are rarely seen, either because of their very small size or because they were not fully mineralized. Except the rays of the anterior dorsal fin, as usually in coelacanths, all the fin rays are slender (characters 108 and 109).

The basal plate of the first dorsal fin is not observed (character 98) in any available specimens. The anterior dorsal fin **(d1.f)** is composed of 15 rays (character 99) (count based on PIMUZ T 1271, 4985 and 5902). The rays are stout, all bearing small and sharp denticles (character 100) (Fig. 2). *Scleracanthus*[18], *Foreyia*[1] and *Allenypterus*[3] have an anterior dorsal fin with also 15 rays, a number situated in the upper range observed in coelacanths. However, compared to *Foreyia*[1], the anterior dorsal fin is shallower in *Rieppelia*.

The basal plate of the posterior dorsal fin **(d2.f.B)** is partially preserved in the holotype (PIMUZ T 5902) (Fig. 2a-b). Its dorsal margin is rounded. Unfortunately, the anteroventral margin of the bone is broken and it is not possible to state if it is simple or forked anteriorly (character 101). The posterior dorsal fin **(d2.f)** is proportionally a very large and plumb lobed fin (Fig. 2). Both anterior and posterior dorsal fins are very large compared to the body size, as in *Foreyia*[1]. There are 38 to 46 rays (count based on PIMUZ T 1271, 4985 and 5902), which is the maximum number of rays observed in coelacanths. Only *Rebellatrix* and *Latimeria* have a posterior dorsal fin with a high number of rays, 33 to 36 and 29 to 31 rays, respectively[10,19].

Indeed, the range of fin rays number in the posterior dorsal fin of coelacanths is usually situated between 14 and 20 rays. Compared to the pelvic and anal fin rays, the rays of the posterior dorsal fin of *Rieppelia* are much elongated, forming then a large and powerful fin. The length of the rays is not symmetrical on both sides of the lobe, the longer fin rays being along the anterior leading edge. Although less marked, this arrangement of the rays in the posterior dorsal fin is also present in *Foreyia*[1].

The basal plate of the anal fin is not preserved in any available specimens. The anal fin **(an.f)** is distinctly a lobed fin, but is relatively small compared to the posterior dorsal fin (Fig. 2). The situation is similar to that of *Foreyia*, which has the anal fin smaller than the posterior dorsal fin[1]. The anal fin of *Rieppelia* includes between 26 to 32 rays (count based on PIMUZ T 1271 and 5902). This number is situated within the upper range observed in the coelacanths. For instance, *Latimeria* and *Rebellatrix* have more rays with 29 to 32 rays and 33 to 36, respectively[10,19], while *Foreyia* has 15 rays in the anal fin. The anal fin of *Rieppelia* inserts posteriorly regarding the second dorsal fin, both fins being then not an exact mirror of each other.

The caudal fin **(cau.f)** is diphycercal (character 104) and is composed of a dorsal and ventral lobes separated by a small supplementary lobe (Fig. 2). As already mentioned, the caudal fin is very large compared to the body size, and forms one third of the total body surface, which is similar to that of *Foreyia*[1]. There is a total of about 30 and 24 rays in the dorsal and ventral lobes, respectively (count based on PIMUZ T 1271 and 5902). With the 13 to 16 dorsal and 11 to 14 ventral radials, the caudal fin shows thus a one-to-two ratio between the radials and the fin rays (character 106). This ratio is known only in some Palaeozoic coelacanths as for instance in *Diplocercides* or *Allenypterus*[3]. According to Forey[3], most coelacanths have two symmetrical lobes with usually one or two fewer rays in the ventral lobe. The dorsal caudal lobes insert on the tail more anteriorly than the ventral caudal lobe giving to the tail a slight asymmetrical appearance. Together with the minimal and maximal count of radials, the caudal tail of *Rieppelia* appears to be then slightly asymmetrical (character 105), unlike to *Foreyia*[1]. Such an asymmetrical caudal tail is observed in some actinistians such as *Laugia*[3] that have 3 to 4 more rays in the dorsal lobe.

The supplementary lobe **(sup.Cau.f.l)** is hardly distinguishable from the dorsal and ventral caudal lobes (Fig. 2). Indeed, the caudal fin has a rounded posterior contour that encloses the supplementary caudal fin lobe, reminiscent of the caudal fin of *Foreyia*[1], *Macropomoides*[3] and potentially *Ticinepomis peyeri*[4]. However, there is small gap between the last posterior radials of both lobes and the anterior most ray of the supplementary lobe, which is composed of 8 rays

(count based on the holotype PIMUZ T 5902). From the posterior last radial to the end of the rays, the supplementary lobe measures an estimated length of 20 mm (by excluding the rays length).

2.8.3 Pectoral girdle and fin

The pectoral girdle is composed of an anocleithrum, a cleithrum, an extracleithrum (character 91) and a clavicle, as in other coelacanths. The scapulocoracoid has not been observed. It is worth noting that in specimen PIMUZ T 1321, the bones of the pectoral girdle are very well preserved in 3D, being then not flattened (Fig. S4).

The anocleithrum **(Ano)** is simple (character 92) and slightly sinusoidal (Fig. 2). Its shape is very similar to the anocleithrum of *Foreyia* (the 'posterior wing of the prootic' of[1]). The bone presents some digitations for the insertion of the muscle that presumably helps to elevate the posterior branchial arches[3].

The cleithrum **(Cl)** is one of the most characteristic bone of *Rieppelia*, which is easily recognizable and often preserved in many specimens (e.g. PIMUZ T 1321, 1639, 1755, 3376 and 5902). It is a large boomerang-shaped bone (Fig. S4). The center of the bone is crossed by a strong lamina that delimitates an anterior and posterior portions of the cleithrum. The anterior portion is interpreted as a branchial lamina **(br.l)** (Fig. S4). It forms a broad medial extension that twists mesially along most the entire length of the cleithrum. The upper border of the lamina, which does not reach the dorsal tip of the cleithrum, is marked by an anterior rounded angle. This lamina is covered by the opercle, whose posterior margin accommodate the central lamina of the cleithrum. Therefore, a large part of the cleithrum is covered by the opercle unlike in most other coelacanths, but as in *Foreyia* that has, however, a thinner cleithrum. This lamina is very similar to the branchial laminae of the cleithrum of some lungfish, such as *Eoctenodus*[20]. The posterior portion of the cleithrum is marked, in its posterorventral lateral surface, by digitation for the insertion of the extracleithrum.

The extracleithrum **(Ecl)** is always observed separated from the cleithrum. It is a bone a little less long than half of the cleithrum. Its distal and dorsal portion is thinner than its proximal portion contacting the clavicle.

The anterior tip of the clavicle **(Cla)**, ovoid shaped, is remarkably smaller than the cleithrum (Fig. S4). From its anterior to its posterior portion, the clavicle twists medially and enlarges dorsally as a thin and large triangular bony portion that was overlapping the ventrolateral part of the cleithrum. The clavicle of *Rieppelia* recalls the clavicle of *Megalocoelacanthus* ([13], fig. 18c), except that in this latter the anterior tip is squarer.

The pectoral fin **(pect.f)** inserts behind the maximal curvature of the cleithrum (Fig. 2). This fin is distinctly large and composed of slender and elongated rays. The fin rays show an asymmetrical profile along the lobe with the longer fin rays located along the anterior leading edge. Compared to the pelvic and anal fins, the pectoral fin is large. There is between 37 and 44 rays in the pectoral fin (count based on PIMUZ T 1271 and 5902), which is the maximum number of rays observed in actinistians, together with *Trachymetopon* having 40 rays[21]. *Foreyia* has only 10 rays in the pectoral girdle, which is one of the lowest number of rays.

2.8.4 Pelvic girdle and fin

The pelvic bones **(P.b)** are remarkably small and thin proportionally to the rest of the skeleton (Fig. 2). The two paired bones are clearly separated (character 103). The pelvic fin **(pelv.f)** lies in abdominal position (character 102) (Fig. 2), as in the majority of actinistians. Although the length of the pelvic fin cannot be observed accurately, the lobe appears to be poorly developed (PIMUZ T 1271 and 5902), which is reminiscent to that of *Foreyia*[1]. The pelvic fin is then of the same size as the anal fin and is considerably smaller than the pectoral fin. The pelvic fin is composed of about 30 rays (count based on PIMUZ T 1271), all with small and sharp denticles. This number of rays is in the upper range for coelacanths, and only *Latimeria*[3] and *Trachymetopon*[21] have more rays with 33 and 35 rays, respectively. The situation is then opposite to *Foreyia* that has only 12 rays in the pelvic fin, a low amount compared to other actinistians[1].

2.9 Ornamentation and histology

2.9.1 Dermal bones

All of the dermal bones – except the gular plates, the oral margin of the premaxillae and the bones of the pectoral girdles – are heavily ornamented with numerous distinct rounded and pointed odontodes (character 28 and 54) (Figs 2a, S1a-b, S2, S3, S6a-b), an ornamentation similar to that of *Foreyia*[1]).

The ornamentation of *Rieppelia* was shortly described by Ørvig[22], and the histology of the dermal bone and the ornaments were thin sectioned and commented by Mutter & Heckert[23]. Thin sections of dermal bones indicate that the ornaments are constituted of a cone of dentine set around a large pulp cavity and overlain by an external hypermineralised layer, representing therefore the typical odontode structure (Fig. S6c-e), a pattern already described by Ørvig[22]. Scales and/or dermal bones having ornaments with the same structural organization is known in other coelacanths such as *Miguashaia*[24], *Undina*[25,26] and *Latimeria*[12,27].

The bony portion on which lies the odontodes is about 600 μm thick (Fig. S6c-e). Within this layer develops a network of vascular cavities and canals **(vc)** (Fig. S6c-e), indicating a high degree of vascularisation[23]. It should be noticed that this layer appears to be highly recrystallised and poorly preserved due to a strong compression of the bones during fossilisation.

Each odontode have a single proportionally and remarkably very large pulp cavity **(pc)** (Fig. S6c-e). It resembles to the large pulp cavity of the odontodes of *Undina*[25,26] or *Latimeria*[12] and is thus larger that the pulp cavity of the odontodes of *Miguashaia*[24].

The dentine layer **(de)** forms the main body of the odontodes and is 200 and 250 μm thick (Fig. S6c-e). From the pulp cavity develop many transversal dentinal tubules within the dentine, representing orthodentine (Mondéjar-Fernández, personal communication 2022). Two zones can be distinguished within the orthodentine layer. There is a first internal thick and bright zone, in which develop the dentinal tubules. The second zone, which surround the latter zone, is almost entirely devoid of tubules. In this zone, there is a succession of thin dark lines intercalated with larger and brighter lines disposed parallel to the external border of the odontodes. The exact nature of those lines remains unknown but they look like lines of arrested of growth.

The external dentine layer is capped by a highly mineralized layer **(en)**, which may possibly represent enamel (Fig. S6c-e). This layer is however very thin, 12 to 14 μm in thickness. The presence of enamel in *Rieppelia* is not surprising as the odontodes ornamenting the scales of, for instance *Latimeria, Undina* and *Miguashaia* are covered by a layer of enamel[12,24-26].

On the dermal bones of *Rieppelia*, the odontodes show a variation of size according to their position on the dermal bones and to the generation to which they belong. Along the crest of the supraorbitals and the opercle (Fig. S4), where the bones angled, the odontodes appear to be slightly larger than those located elsewhere.

In PIMUZ T 1638 (Fig. S6a-b), displaying a very well-preserved ornamentation, there is clearly two generations of odontodes, with small and low odontodes located between large and high odontodes. The presence of different generations of odontodes representing different stages of growth was already observed by Ørvig ([22], plate 2A). Superimposed odontodes is not obviously observed and most odontodes grow next to other odontodes (Fig. S6a). Few odontodes, however, appear to grow just above the marginal border of a neighbouring odontode (Fig. S6b). The situation in *Rieppelia* is thus definitively different from *Miguashaia* ([24], figs 3a and 4a) or *Spermatodus* ([28], fig. 1.3) that both have strongly superimposed generation of odontodes. As there is no obvious superimposed odontodes, it is unclear if there is a fixed

number of generations of odontodes or if odontodes are growing constantly replacing either older or lost odontodes.

2.9.2 Scales

The holotype (PIMUZ T 5902) is the only specimen that has preserved the scales in situ on the body (Fig. 2a-b). The scales are subcircular to suboval ([29], fig 3) (Fig. S7a-b) as in *Foreyia* ([1], figs 2, S2) and *Diplocercides* and unlike *Latimeria*[24], for instance. The scales show some variations of their ornamental pattern according to their position on the body.

The anterior portion of each scale is largely overlapped by the preceding scales. In this area, there are several concentric rings of growth. The exposed area is ornamented with numerous blunt spines and represents approximately one third of the area of the scale (Fig. S7a-c). There are about 25 blunt spines aligned on an anteroposterior axis and arranged in 5 to 6 rows, as already described by Rieppel[29]. The spines of the posterior most rows extend beyond the posterior margin of the scale. According to Rieppel[29], the spines lack dentine. Unfortunately, no histological study was made to support this observation but his affirmation may be questioned because the histology of the dermal bones presents a highly mineralised layer, probably made of enamel. Indeed, it seems that the histological structure is similar between the dermal bones and the scales as exemplified by *Miguashaia*[24] and *Latimeria*[27]. Therefore, it would be surprising if the situation was not similar in *Rieppelia*.

Regarding the position of the scales on the body of the holotype (PIMUZ T 5902), the size of the spines appears to vary very slightly in size (Fig. S7c). On the upper part of the flank and anteriorly to the first dorsal fin, the spines are larger and longer than on the rest of the body. On the anal fin, the spines appear to be relatively small and short. Although the length of the spines varies from a scale to another, there is no variation of length of the spines within a single scale meaning they are not differentiated (character 112). Due to the preservation, it is hard to distinguish the condition of the openings for the lateral lines within the scales because of the preservation (character 110).

In the most ventral portion of the body (PIMUZ T 5902), there are some round to ovoid thick scales (Fig. 2a, not labelled and located below and around the possible basibranchial). Those scales bear in their center between 10 to 15 small and low ornament with a round apex resembling to hemispherical bulges (Fig. S7d-e). The external rim of those scales is covered with many thin radiating ridges. When flipped in internal view, the scales display small depression in their center corresponding to bulges on the external aspect (PIMUZ T 5902). These peculiar scales are reminiscent to the ventral-most scales of the belly of *Foreyia*, except

that in the latter taxon there are only two to four spines ([1], fig. 2c6). As in *Foreyia*, these peculiar scales may have formed a paving-like structure that would have act as an armoured protection on the belly of *Rieppelia*. It should be warned that in both taxa, these scales are not comparable to the ventral keel scales (character 111) described in few coelacanths, such as in *Allenypterus*[3].

3 Supplementary Figures

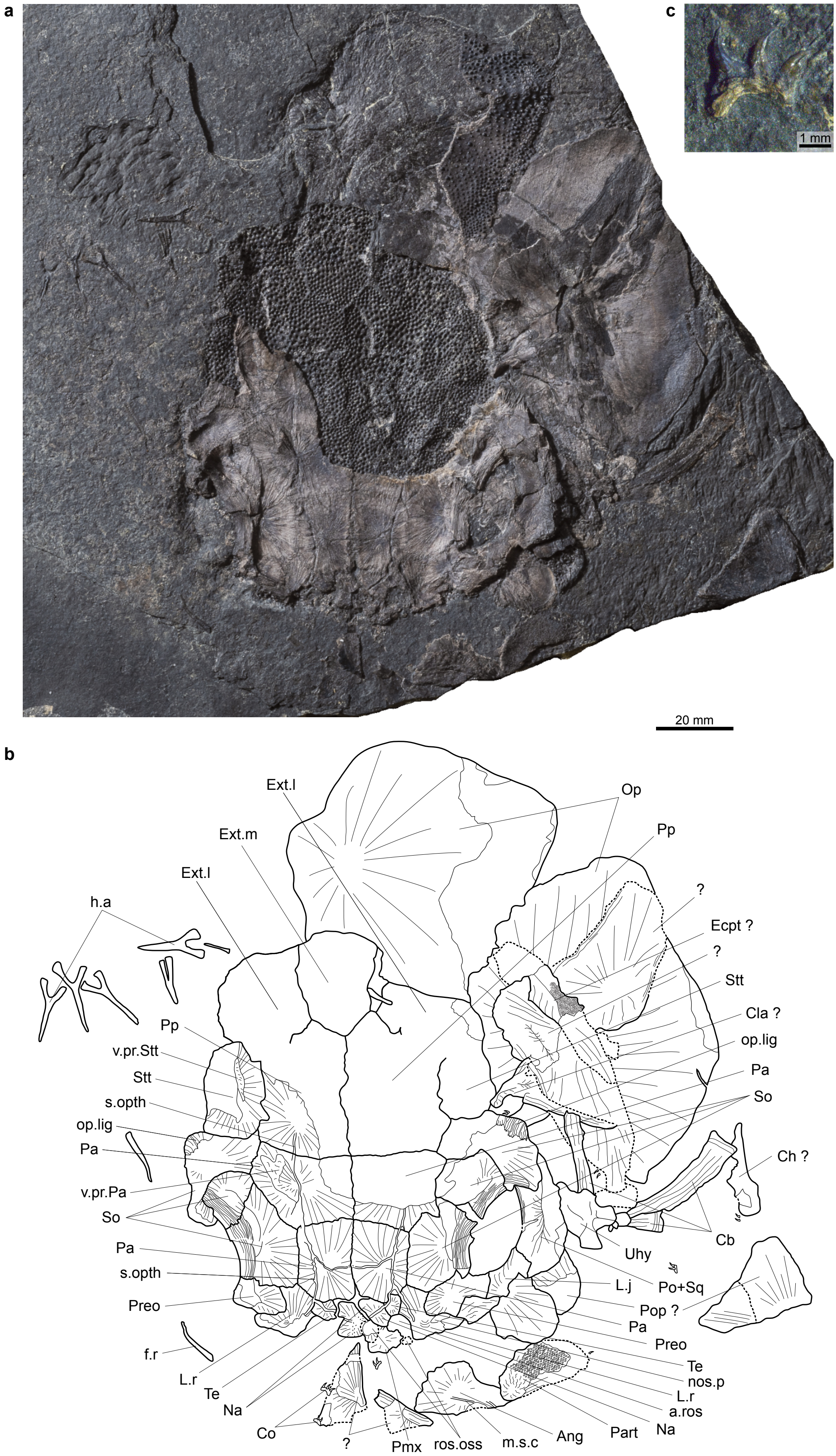

Figure S1 ***Rieppelia heinzfurreri*** **gen. et sp. nov. a**, Photograph and **b**, drawing of the counterpart of the paratype PIMUZ T 1638b showing the dermal bones of the skull and other bones partly in internal view. c, Enlargement of one of the coronoids showing the cluster of three tiny curved teeth bound on a bony base. Abbreviations: a.ros, anterior opening for the rostral organ; Ang, angular; Cb, ceratobranchial; Ch, ceratohyal; Co, coronoid; Ecpt, ectopterygoid; Ext.l, lateral extrascapular; Ext.m, median extrascapular; f.r, fin ray; h.a, haemal arches; L.j, Lachrymojugal; L.r, lateral rostral; m.s.c, mandibular sensory canal; Na, nasal; nos.p, posterior nostril; Op, opercle; op.lig, insertion point for opercular ligament; Pa, parietal; Part, prearticular; Pmx, premaxilla; Po+Sq, postorbital + squamosal; Pop, preopercle; Pp, postparietal; Preo, preorbital; Q, quadrate; ros.oss, rostral ossicles; s.opth, superficial ophthalmic nerve (anterodorsal lateral line nerve); So, supraorbital; Stt, supratemporal; Te, tectal; Uhy, urohyal; v.pr.Pa, ventral descending process of the parietal; v.pr.Stt, ventral descending process of supratemporal.

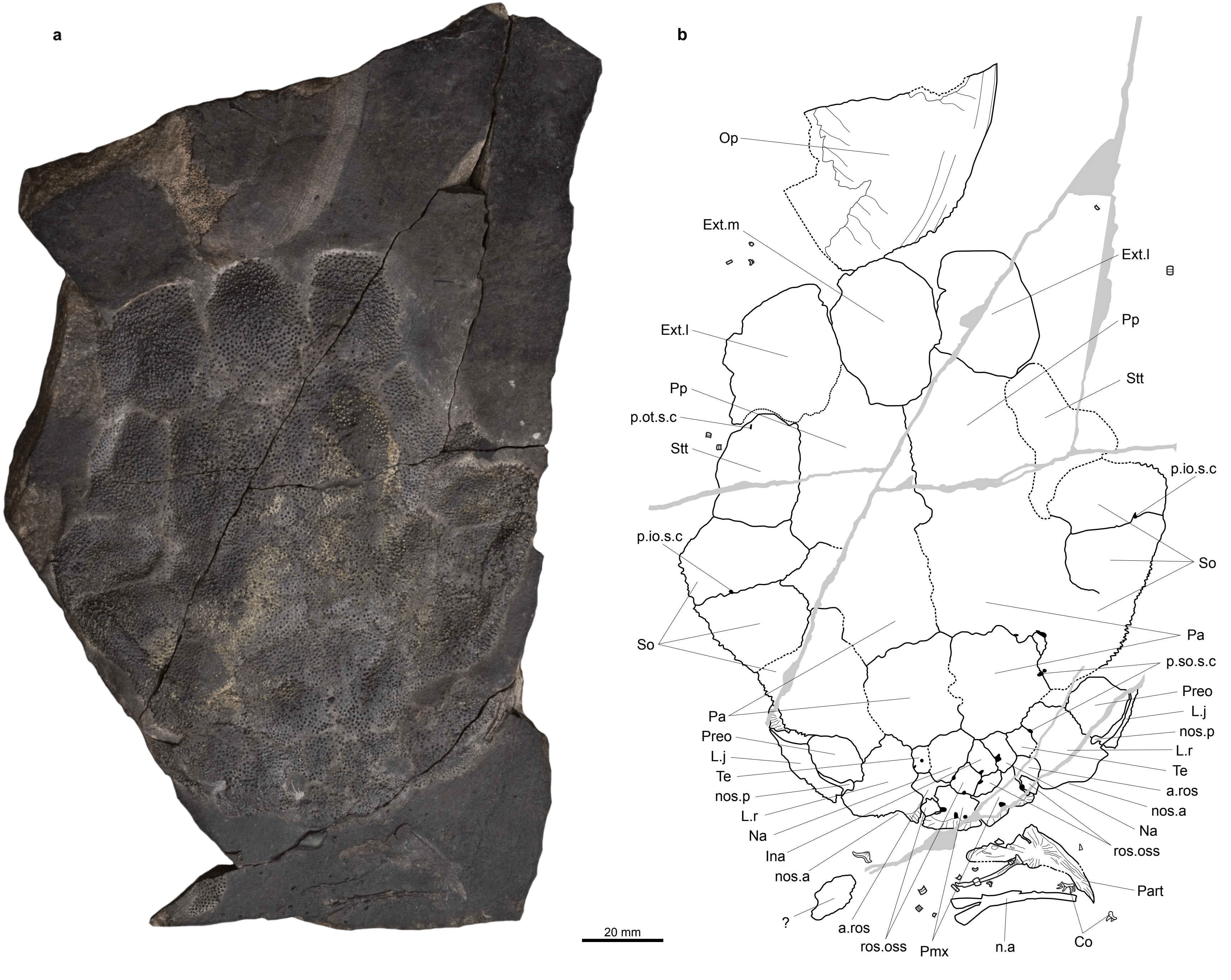
a
b
Op
Ext.m
Ext.l
Pp
Stt
p.ot.s.c
p.io.s.c
So
Pa
p.so.s.c
Preo
L.j
nos.p
L.r
Te
a.ros
nos.a
Na
ros.oss
Ina
Part
?
Pmx
n.a
Co
20 mm

Figure S2 ***Rieppelia heinzfurreri*** **gen. et sp. nov. a**, Photograph and **b**, drawing of the paratype PIMUZ T 5905 showing the skull roof in dorsal view and elements of the lower jaw. Abbreviations: a.ros, anterior opening for the rostral organ; Co, coronoid; Ext.l, lateral extrascapular; Ext.m, median extrascapular; Ina, internasal; L.j, Lachrymojugal; L.r, lateral rostral; n.a, neural arches; Na, nasal; nos.a, anterior nostril; nos.p, posterior nostril; Op, opercle; p.io.s.c, pore for the infraorbital sensory canal; p.ot.s.c, pore for the otic sensory canal; p.so.s.c, pore for the supraorbital sensory canal; Pa, parietal; Part, prearticular; Pmx, premaxilla; Pp, postparietal; Preo, preorbital; ros.oss, rostral ossicle; So, supraorbital; Stt, supratemporal; Te, tectal.

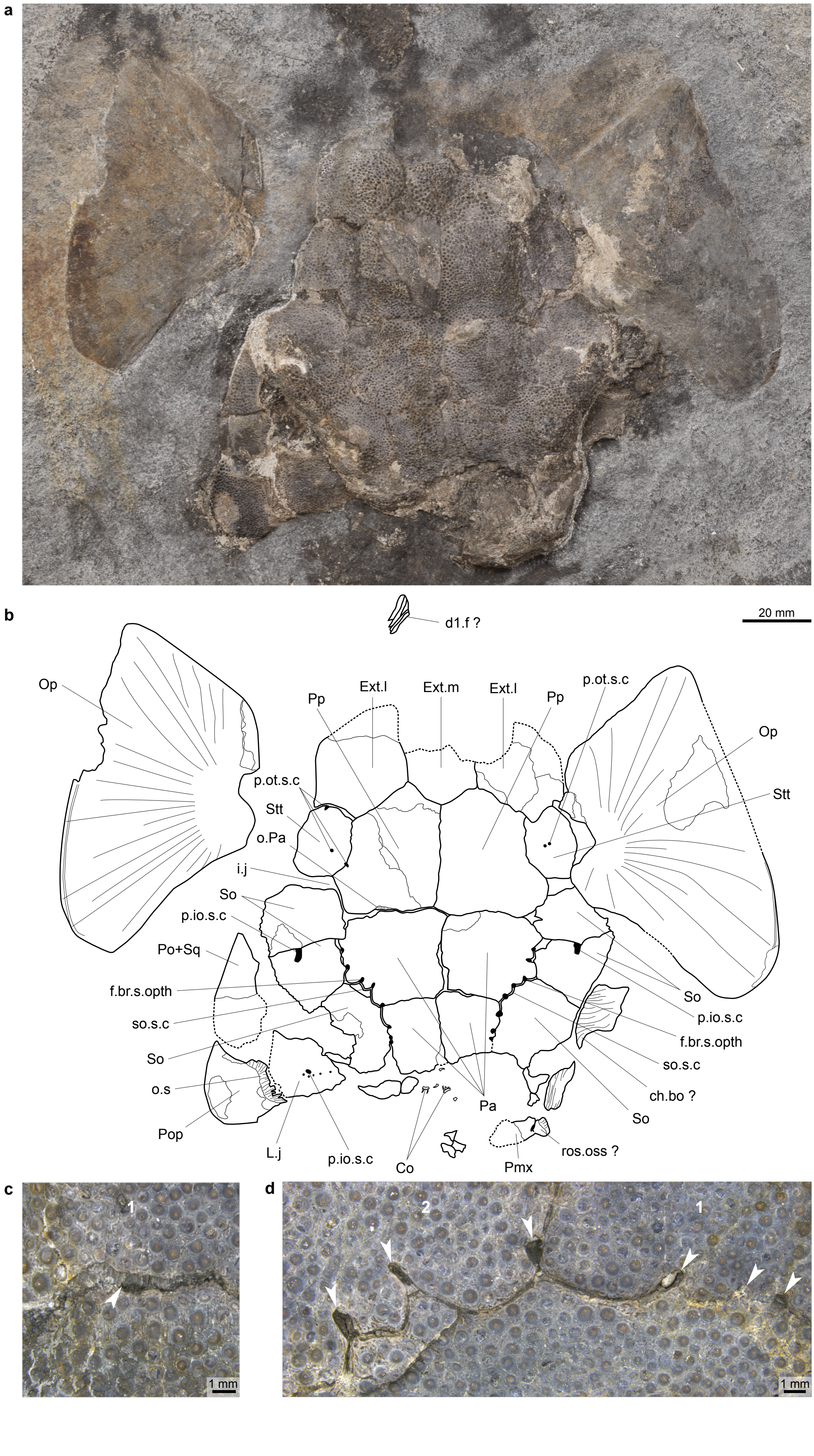
a
b
d1.f ?
20 mm
Op
Pp
Ext.l
Ext.m
Ext.l
Pp
p.ot.s.c
Op
p.ot.s.c
Stt
Stt
o.Pa
i.j
So
p.io.s.c
Po+Sq
f.br.s.opth
so.s.c
So
o.s
Pop
L.j
p.io.s.c
Co
Pa
Pmx
ros.oss ?
So
ch.bo ?
so.s.c
f.br.s.opth
p.io.s.c
So
c
1
1 mm
d
2
1
1 mm

Figure S3 ***Rieppelia heinzfurreri* gen. et sp. nov. a**, Photograph and **b**, drawing of specimen PIMUZ T 5903 showing the skull roof, the cheek and the opercles. **c**, Enlargement of the intracranial joint at the level of the right postparietal (1) showing the areas of overlaps (white arrowhead) for the posterior parietal. **d**, Enlargement of the supraorbital sensory canal that runs across the anterior (1) and posterior (2) right parietals showing the foramens (white arrowhead) for branches of the superficial ophthalmic branch of the anterodorsal lateral nerve. Abbreviations: Co, coronoid; d1.f, anterior dorsal fin; Ext.l, lateral extrascapular; Ext.m, median extrascapular; f.br.s.opth, foramen for the superficial ophthalmic nerve; i.j, intracranial joint; L.j, Lachrymojugal; o.Pa, overlap surface for posterior parietal; o.s, overlap surface; Op, opercle; p.io.s.c, pore for the infraorbital sensory canal; p.ot.s.c, pore for the otic sensory canal; Pmx, premaxilla; Po+Sq, postorbital + squamosal; Pop, preopercle; Pp, postparietal; ros.oss, rostral ossicle; So, supraorbital; so.s.c, supraorbital sensory canal; Stt, supratemporal.

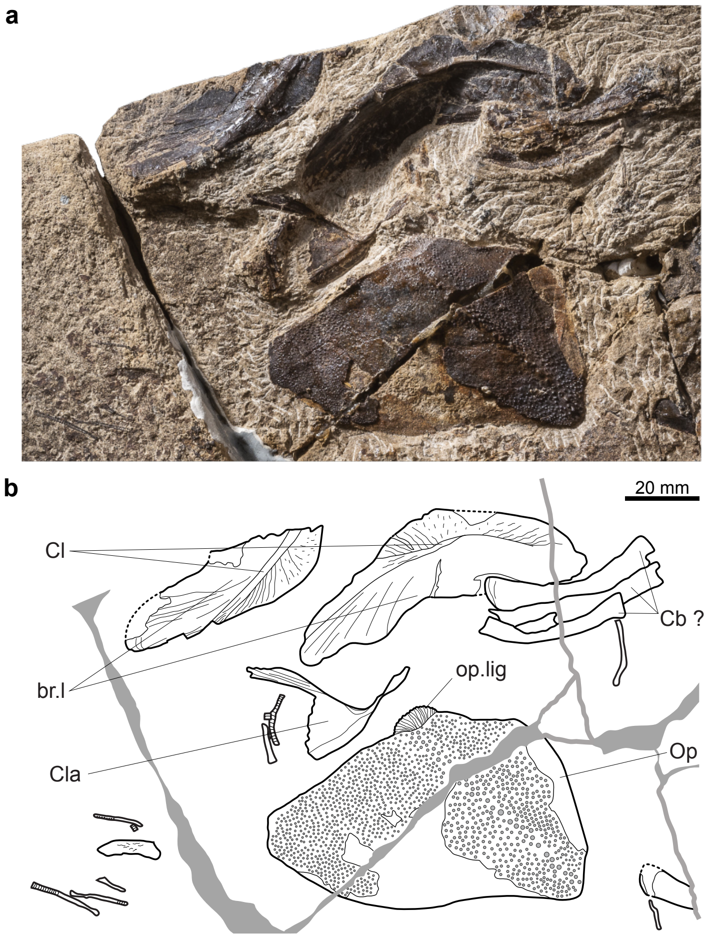

Figure S4 ***Rieppelia heinzfurreri*** **gen. et sp. nov. a**, Photograph and **b**, drawing of specimen PIMUZ T 1321 showing the left opercle and elements of the pectoral girdle. It is worth noting that the bones of this specimen have been poorly compressed during fossilisation. Abbreviations: Cl, cleithrum; br.l, branchial lamina of cleithrum; Cb, ceratobranchial; Cla, clavicle; Op, opercle; op.lig, insertion point for opercular ligament.

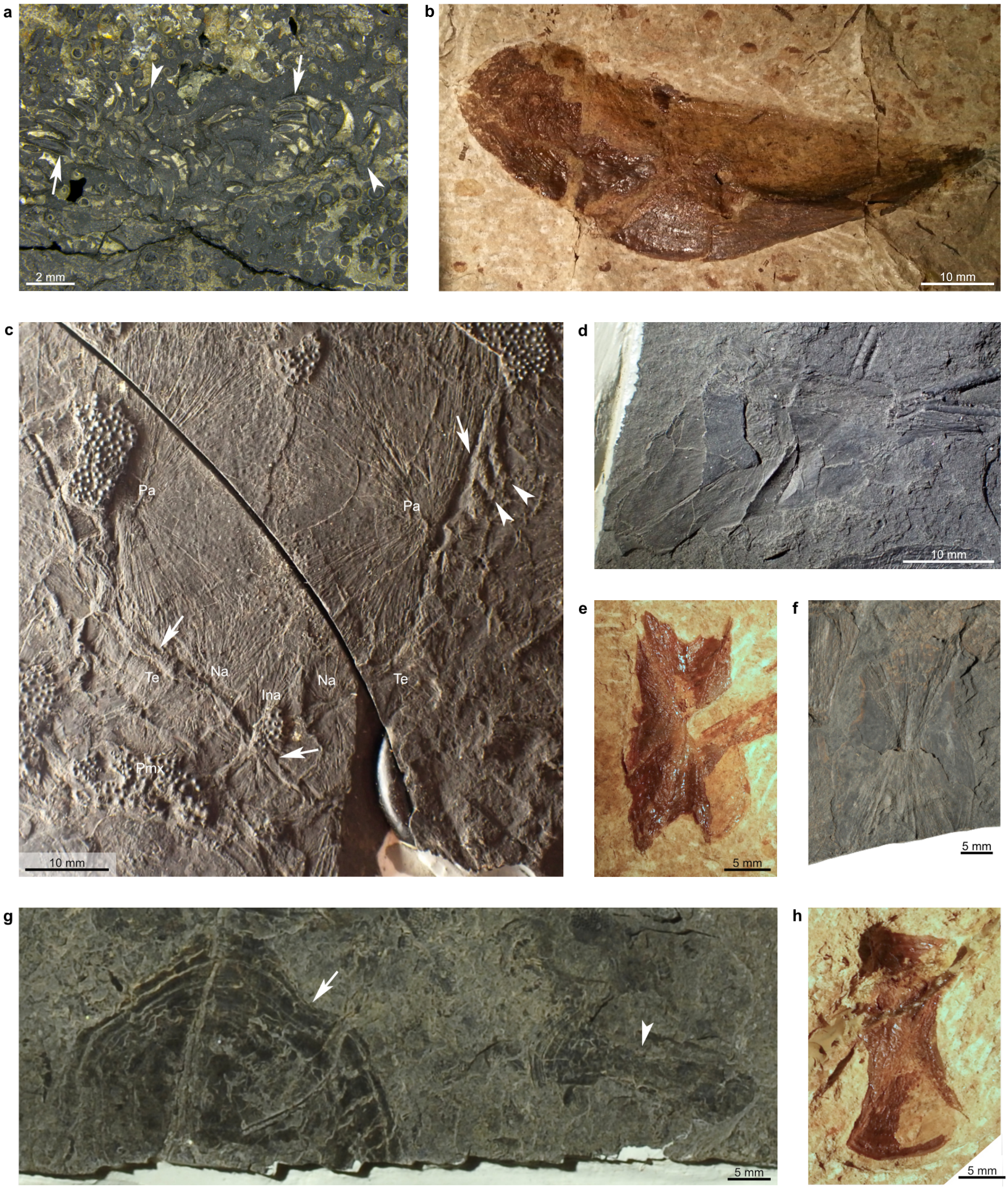

Figure S5 Additional isolated bones and anatomical features of *Rieppelia heinzfurreri* gen. et sp. nov. a, Coronoids (white arrowhead) and shedded teeth of the dentary tooth plates (white arrow) as preserved above the right lower jaw of the holotype PIMUZ T 5902. **b**, Gular plate (PIMUZ T 1273). **c**, Postparietal shield of PIMUZ T 1755 showing a 3D imprint of the groove interpreted as the path of the superficial ophthalmic nerve (white arrow) with lateral directed branches (white arrowhead) that may correspond to the branches innervating the supraorbital

sensory canal. In the snout area in front of the internasal and nasals, the groove form a loop (the structure may represent both the superficial ophthalmic nerve and the sensory canal, which have been compressed together during fossilisation). **d**, Isolated pterygoid (PIMUZ T 1755). **e** and **f**, Isolated urohyal (PIMUZ T 1273 and 1632, respectively). **g**, Isolated ossified hyomandibular (white arrow) and symplectic (white arrowhead) (PIMUZ T 1755). **h**, isolated symplectic (PIMUZ T 1273). Abbreviations: Ina, internasal; Na, nasal, Pa, anterior parietal; Te, tectal.

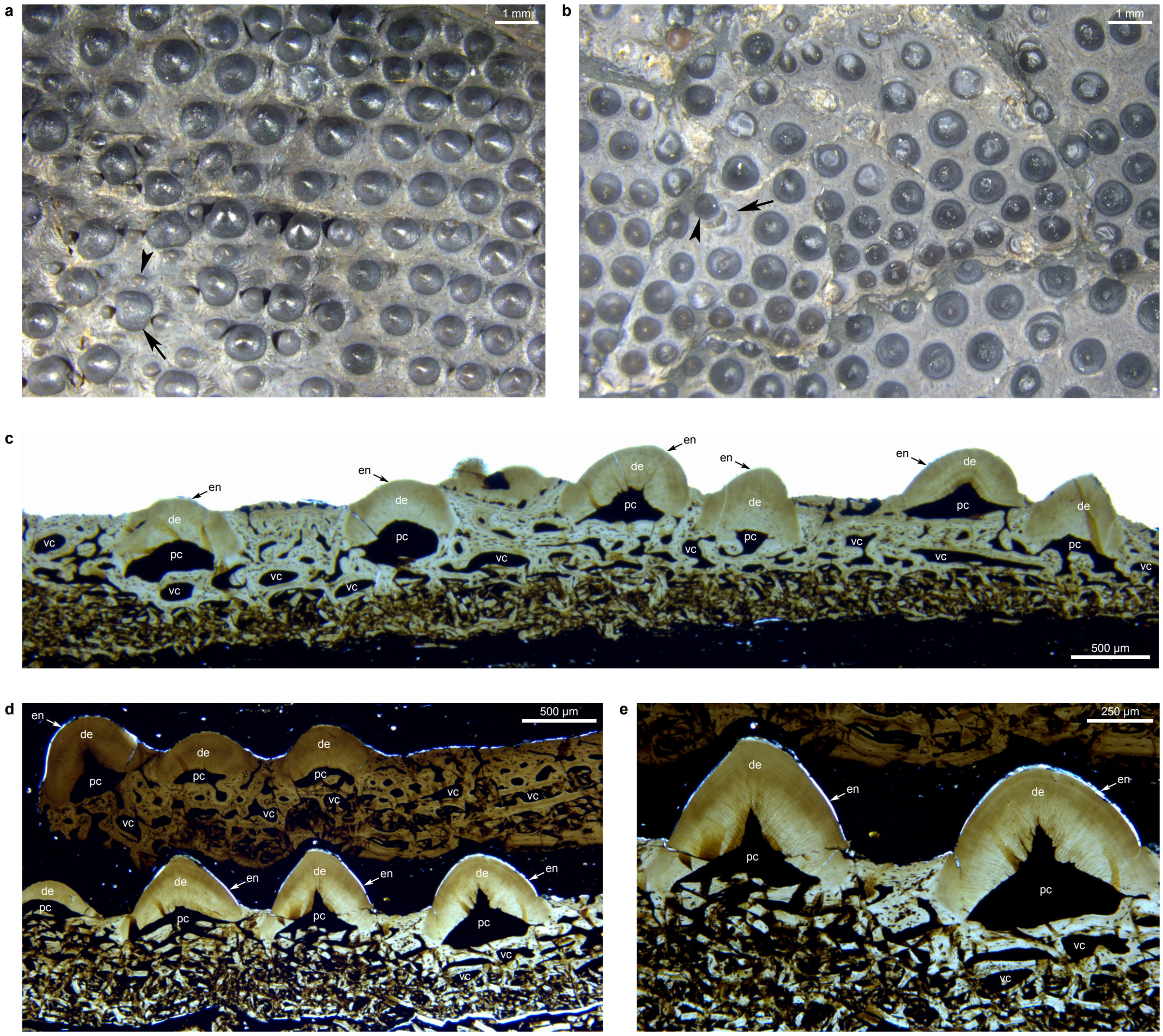

Figure S6 Ornamentation and microstructural organisation of the dermal bones of *Rieppelia heinzfurreri* gen. et sp. nov. a, External surface of a dermal bone of the skull roof displaying numerous odontodes of two different generations. Small odontodes, representing a new generation of odontodes (dark arrwohead), growth around larger odontodes, which represent an older generation (dark arrow). **b**, External surface of a dermal bone of the skull roof displaying numerous odontodes. After an odontode is lost (dark arrow), a neighbouring odontode may grow above the socket of the lost odontode (dark arrowhead). **c**, Thin section of an undetermined dermal bone with odontodes. **d**, Thin section of two undetermined dermal bones with odontodes. **e**, Enlargement of d. The pulp cavity is very large proportionally to the odontode. The dentine layer appears to be structured by an internal bright layer overlapped by a darker external layer. Many transversal dentinal tubules are sent from the pulp cavity into the internal dentine layer. The dentine layer represents then orthodentine. The external layer displays an alternance of many thin bright and dark layers developing parallel to the external

rim of the odontode. The external rim of the odontode is capped by a very thin layer of a highly mineralised material. Abbreviations: de, dentine; en, highly mineralised layer; pc, pulp cavity; vc, vascular canal.

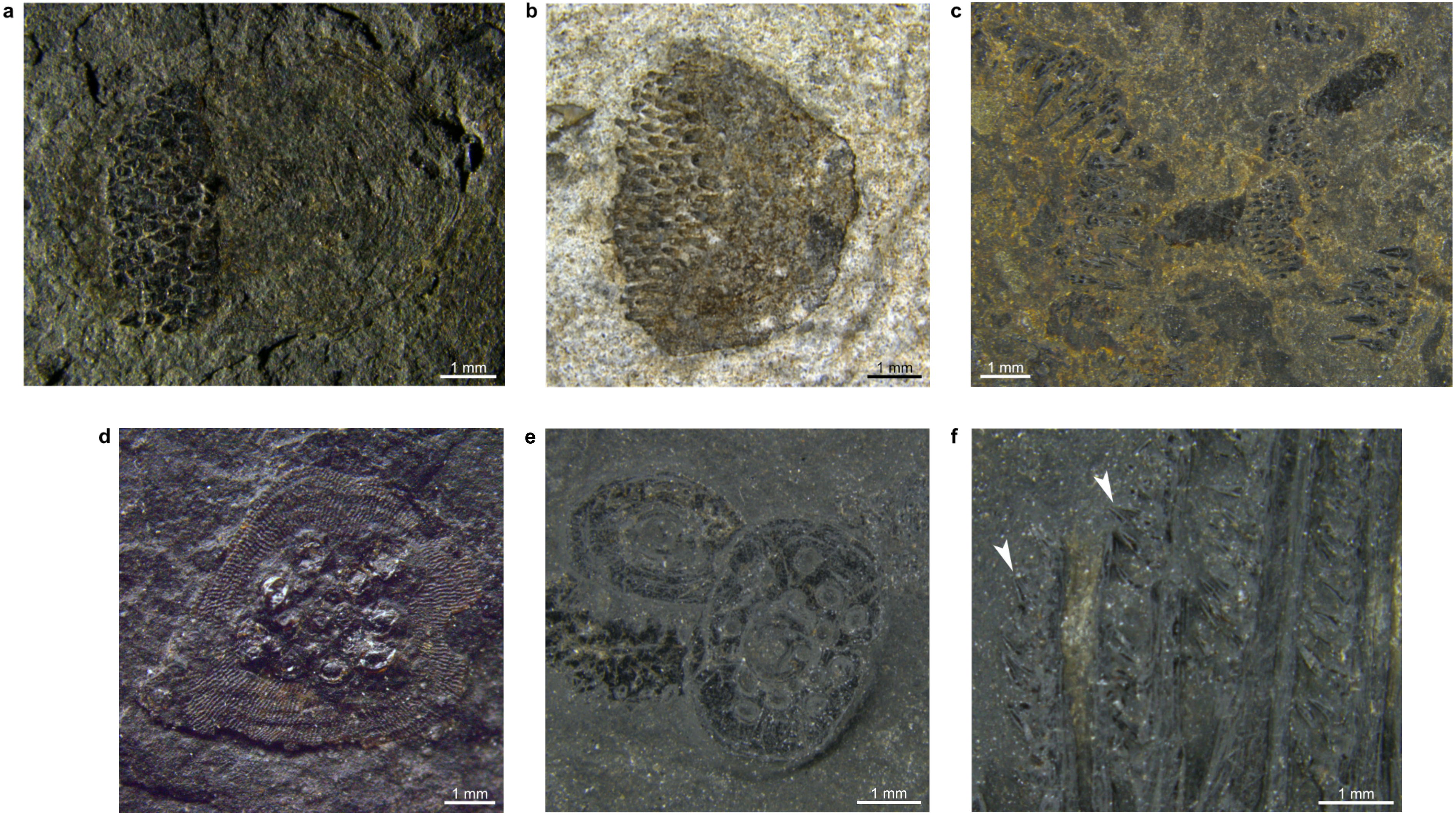

a
1 mm
b
1 mm
c
1 mm
d
1 mm
e
1 mm
f
1 mm

Figure S7 Scales and ornamentation of fin rays of *Rieppelia heinzfurreri* gen. et sp. nov. a, Isolated ovoid scale (PIMUZ T 5906). **b**, Incomplete imprint of an isolated scale (PIMUZ T 4985). **c**, Exposed portions of scales (PIMUZ T 1639) showing variation in size of the denticles. **d**, Isolated ovoid scale from the belly (PIMUZ T 5907). The scale is ornamented in its centre by 15 small and low ornament with a round apex resembling to hemispherical bulges. The external rim is covered with many thin radiating ridges. **e**, Isolated ovoid scale from the belly in internal view (holotype, PIMUZ T 5902). **f**, Denticles (white arrowheads) ornamenting the rays of the upper lobe of the caudal fin (holotype, PIMUZ T 5902).

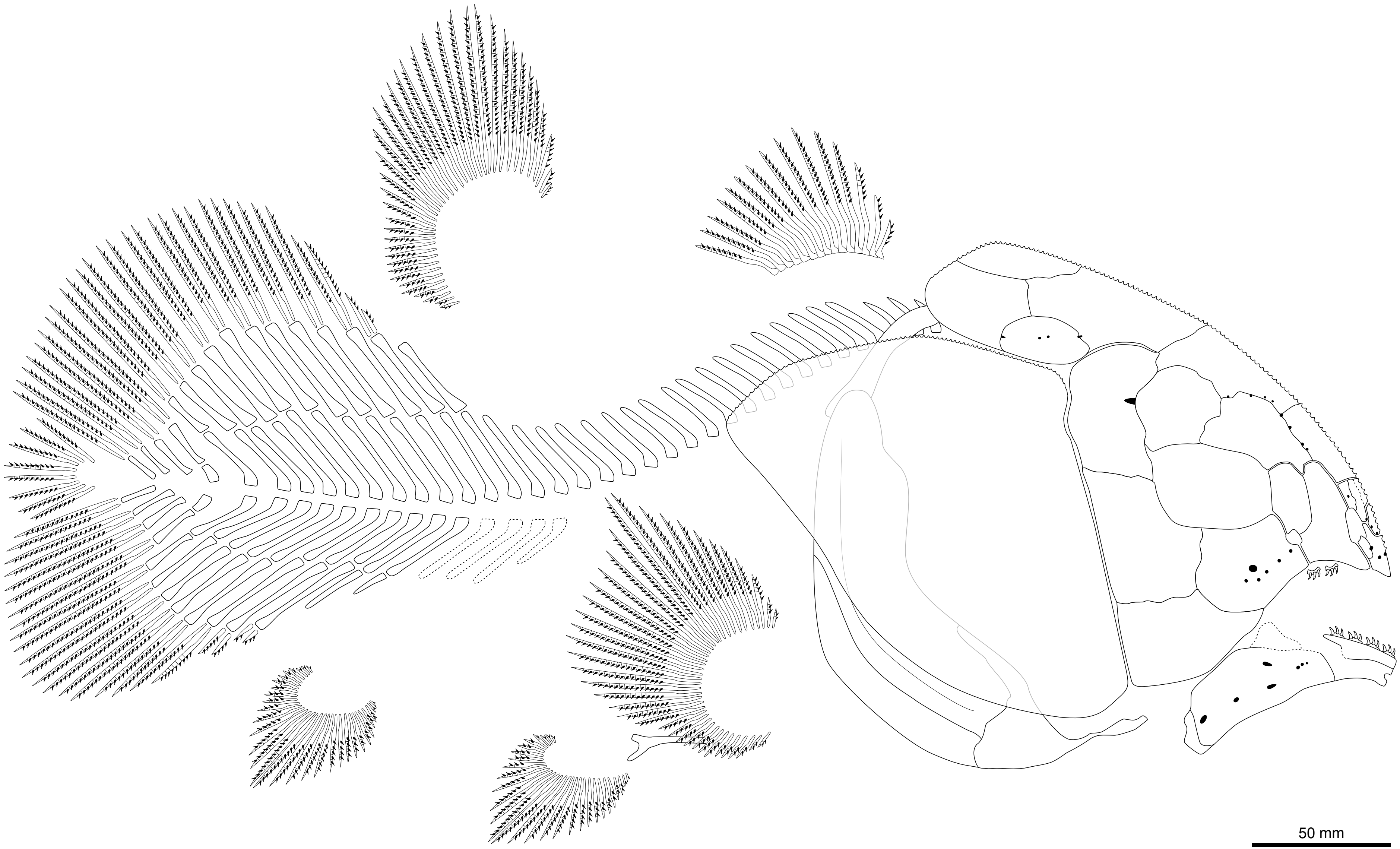

Figure S8 Reconstruction of *Rieppelia heinzfurreri* gen. et sp. nov.

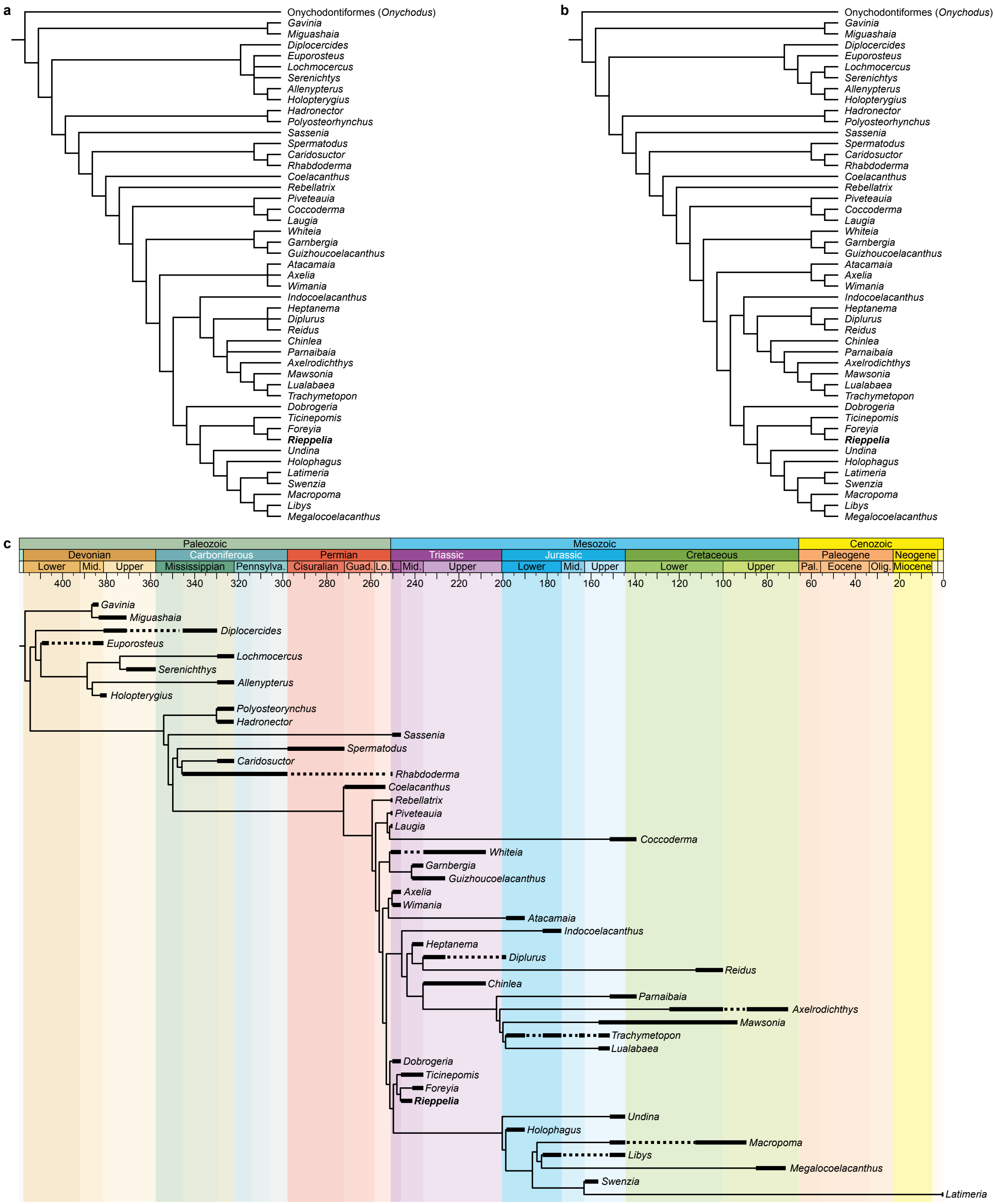

Figure S9 Phylogenetic analysis. a, Strict consensus tree of 135 most parsimonious tree, unweighted. **b**, One of the 135 most parsimonious trees; Tree length = 310; Consistency index = 0.397; Homoplasy index = 0.603; Retention index = 0.701; Rescaled consistency index = 0.278. **c**, Time-tree phylogeny of Actinistia, plotted against the International Chronostratigraphic Chart.

4 Phylogenetical analyses

4.1 List of characters

Dermal bones of the skull roof

Character 1

Parietonasal and postparietal shields [New character]

0. free from one to another
1. sutured to each other

Character 2

Parietonasal versus postparietal shields [New character]

0. smaller or almost of the same length
1. longer

Character 3

Snout bones [Forey 1998: character 2]

0. lying free from one another
1. consolidated

Character 4

Premaxillary teeth [New character]

0. equal or more than 5
1. equal or less than 4

Character 5

Premaxilla [Forey 1998: character 5]

0. with dorsal lamina
1. without dorsal lamina

Character 6

Anterior opening of the rostral organ [Forey 1998: character 6]

0. contained within premaxilla
1. within separated rostral ossicles

Character 7

Internasal [New definition of character 3 of Forey 1998]

0. several
1. one or none

Character 8

Parietal [Forey 1998: character 7]

0. one pair
1. two pairs

Character 9

Anterior and posterior pairs of parietals [Forey 1998: character 8]

0. of similar size
1. of dissimilar size

Character 10

Parietals and postparietals [Forey 1998: character 28]

0. without raised areas
1. with raised areas

Character 11

Parietal descending process [New definition of character 11 of Forey 1998]

0. absent or highly reduced to a ridge
1. present

Character 12

Number of supraorbitals/tectals [New definition of character 9 of Forey 1998]

0. equal or less than 9
1. equal or more than 10

Character 13

Preorbital [Forey 1998: character 10]

0. absent
1. present

Character 14

Intertemporal [Forey 1998: character 12]

0. absent
1. present

Character 15

Postparietal descending process [New definition of character 13 of Forey 1998]

0. absent or highly reduced to a ridge
1. present

Character 16

Supratemporal descending process [New definition of character 14 of Forey 1998]

0. absent or highly reduced to a ridge
1. present

Character 17

Posterior margin of the skull roof [Forey 1998: character 18]

0. straight
1. embayed

Character 18

Extrascapulars [Forey 1998: character 15]

0. sutured with postparietals
1. free

Character 19

Extrascapulars [Forey 1998: character 16]

0. behind level of neurocranium
1. forming part of the skull roof

Character 20

Pair(s) of lateral extrascapulars (without the triple junction for sensory canals) [New definition of character 17 of Forey 1998]

0. none
1. one
2. two or more

Character 21

Median extrascapular [New character]

0. present
1. absent

Character 22

Supraorbital sensory canal [Forey 1998: character 19]

0. running through centre of ossification
1. following sutural course

Character 23

Supraorbital sensory canals opening as [New definition of character 23 of Forey 1998]

0. few pores at the sutural contact of bones
1. bifurcating pores
2. many pores within bones
3. continuous groove crossed by pillars
4. continuous groove without pillars

Character 24

Medial branch of otic canal [Forey 1998: character 20]

0. absent
1. present

Character 25

Anterior branches of supratemporal commissure [Forey 1998: character 22]

0. absent
1. present

Character 26

Pit lines [Forey 1998: character 26]

0. marking postparietals
1. not marking postparietals

Character 27

Middle and posterior pit lines [Forey 1998: character 25]

0. within posterior half of postparietals
1. within anterior third

Character 28

Dermal bones of the skull roof ornamented with [New definition of character 27 of Forey 1998]

0. coarse and/or irregularly shaped tubercles and/or elongated continuous/discontinuous vermiform/linear ridged tuberculation
1. round tubercles
2. coarse rugosities and fine to pronounced striae
3. mostly or entirely unornamented

Cheek bones and sensory canals

Character 29

Cheek bones [Forey 1998: character 29]

0. sutured to one another
1. separated from one another

Character 30

Spiracular (postspiracular) [Forey 1998: character 30]

0. absent
1. present

Character 31

Postorbital [Forey 1998: character 40]

0. simple, without anterodorsal excavation
1. anterodorsal excavation in the postorbital

Character 32

Postorbital [Forey 1998: character 41]

0. without anterior process
1. with anterior process

Character 33

Postorbital [Forey 1998: character 42]

0. large
1. reduced to a narrow tube surrounding the sensory canal only

Character 34

Postorbital [Forey 1998: character 43]

0. entirely behind the level of the intracranial joint

1. spanning the intracranial joint

Character 35

Jugal [New character]

0. present

1. absent

Character 36

Squamosal [Forey 1998: character 37]

0. large

1. reduced to a narrow tube surrounding the jugal sensory canal only

Character 37

Squamosal [Forey 1998: character 34]

0. limited to the mid-level of cheek

1. extending behind the postorbital to reach the skull roof

Character 38

Preopercle [Forey 1998: character 38]

0. large

1. reduced to a narrow tube surrounding the preopercular canal only

Character 39

Preopercle [Forey 1998: character 39]

0. undifferentiated

1. developed as a posterior tube-like canal-bearing portion and an anterior blade-like portion

Character 40

Position of the preopercle within the cheek [New character]

0. posterior to the squamosal and/or the postorbital

1. below or anterior to the squamosal and the postorbital

Character 41

Subopercle [Forey 1998: character 32]

0. absent

1. present

Character 42

Anterior end of the lachrymojugal [New character; based on characters 35 and 36 of Forey 1998]

0. simple
1. angled and/or expanded

Character 43

Lachrymojugal [New character]

0. with parallel margins along its entire lenght
1. with a more or less thick triangular portion

Character 44

Contact between the lachrymojugal and the preorbital or tectal-supraorbital series [Cavin et al. 2017: character 51]

0. present
1. absent

Character 45

Posterior nostril on the lachrymojugal [New character]

0. not marked
1. marked

Character 46

Posterior opening of the rostral organ marks [New character]

0. the preorbital
1. the lachrymojugal
2. the tectal and/or no bones

Character 47

Posterior opening(s) of the rostral organ mark(s) bone as [New character]

0. foramen(s)
1. notch(es) or groove(s)
2. not marking bone

Character 48

Anterior and/or posterior branches of the infraorbital canal within the postorbital [New definition of character 44 of Forey 1998]

0. absent (canal simple)
1. present

Character 49

Infraorbital sensory canal [Forey 1998: character 45]

0. running through centre of postorbital
1. running at the anterior margin of the postorbital

Character 50

Prominent branches of the jugal sensory canal within the squamosal [New definition of character 46 of Forey 1998]

0. absent (canal simple)
1. present

Character 51

Jugal sensory canal [Forey 1998: character 47]

0. running through centre of bone
1. running along the ventral margin of the squamosal

Character 52

Infraorbital, jugal and preopercular sensory canals [Dutel et al. 2012: character 50]

0. opening through many tiny pores
1. opening through a few large pores
2. opening as a large, continuous groove crossed by pillars

Character 53

Pit lines [Forey 1998: character 48]

0. marking cheek bones
1. failing to mark cheek bones

Character 54

Dermal bones of the cheek ornamented with [New definition of character 49 of Forey 1998]

0. coarse and/or irregularly shaped tubercles and/or elongated continuous/discontinous vermiform/linear ridged tuberculation
1. round tubercles
2. coarse rugosities and fine to pronounced striae
3. mostly or entirely unornamented

Character 55

Orbital space [New character]

0. small and occupied entirely by the eye
1. large and not entirely occupied by the eye

Character 56

Sclerotic ossicles [Forey 1998: character 52]

0. absent
1. present

Lower jaw

Character 57

Retroarticular and articular [Forey 1998: character 53]

0. co-ossified
1. separated

Character 58

Dentary [Forey 1998: character 57]

0. simple
1. dentary hook-shaped

Character 59

Dentary [Forey 1998: character 65]

0. without prominent lateral swelling
1. with swelling

Character 60

Dentary [New definition of character 54 of Forey 1998]

0. with fused dentary teeth
1. with separated dentary teeth or edentulous

Character 61

Principal coronoid [Forey 1998: character 66]

0. lying free
1. sutured to angular

Character 62

Number of anterior coronoids [New definition of character 55 of Forey 1998]

0. four or more
1. three or less

Character 63

Coronoid [Forey 1998: character 56]

0. opposite to the posterior end of dentary not modified
1. modified

Character 64

Coronoid fangs [Forey 1998: character 67]

0. absent
1. present

Character 65

Prearticular and/or coronoid teeth [Forey 1998: character 68; New definition of character 68 of Wen et al. 2013]

0. pointed and smooth
1. rounded and marked with fine striations radiating from the crown

Character 66

Subopercular branch of the mandibular sensory canal [Forey 1998: character 60]

0. absent
1. present

Character 67

Dentary sensory pore [Forey 1998: character 61]

0. absent
1. present

Character 68

Mandibular sensory canal on the splenial [New character]

0. opening through laterally directed pores
1. opening through ventrally directed pores

Character 69

Oral pit line [New character]

0. marking the angular
1. not marking the angular

Character 70

Oral pit line [Forey 1998: character 58]

0. confined to angular
1. oral pit line reaching forward to the dentary and/or the splenial

Neurocranium, parasphenoid and vomer

Character 71

Orbitosphenoid and basisphenoid regions [Forey 1998: character 69]

0. co-ossified
1. separate

Character 72

Processus connectens [Forey 1998: character 71]

0. failing to meet parasphenoid
1. meeting parasphenoid

Character 73

Basipterygoid process [Forey 1998: character 72]

0. absent
1. present

Character 74

Temporal excavation [Forey 1998: character 74]

0. not lined with bone
1. lined with bone

Character 75

Otico-occipital [Forey 1998: character 75]

0. solid
1. separated to prootic/opisthotic

Character 76

Supraoccipital [Forey 1998: character 76]

0. absent
1. present

Character 77

Toothed area of the parasphenoid [New character]

0. covers most of the ventral surface
1. restricted to the anterior half

Character 78

Buccohypophysial canal [Forey 1998: character 78]

0. closed
1. opening through parasphenoid

Character 79

Parasphenoid [Forey 1998: character 79]

0. without ascending laminae anteriorly
1. with ascending laminae

Character 80

Suprapterygoid process [Forey 1998: character 80]

0. absent
1. present

Character 81

Vomers [Forey 1998: character 81]

0. not meeting in the midline
1. meeting medially

Character 82

Prootic [Forey 1998: character 82]

0. without complex suture with the basioccipital
1. with a complex suture

Character 83

Superficial ophthalmic branch of anterodorsal lateral line nerve [Forey 1998: character 83]

0. not piercing antotic process
1. piercing antotic process

Character 84

Process on braincase for articulation of infrabranchial 1 [Forey 1998: character 84]

0. absent

1. present

Character 85

Separate lateral ethmoids [Forey 1998: character 85]

0. absent

1. present

Character 86

Separate basioccipital [Forey 1998: character 86]

0. absent

1. present

Character 87

Dorsum sellae [Forey 1998: character 87]

0. small

1. large and constricting entrance to cranial cavity anterior to the intracranial joint

Palate, Hyoid and gill arches

Character 88

Ventral swelling of the palatoquadrate [Dutel et al. 2012: character 110]

0. absent

1. present

Character 89

Basibranchial tooth plates [New character]

0. three median pairs or more

1. two median pairs or less

Character 90

Anterior basibranchial tooth plates [New character]

0. paired

1. fused

Postcranial skeleton

Character 91

Extracleithrum [Forey 1998: character 88]

0. absent
1. present

Character 92

Anocleithrum [Forey 1998: character 89]

0. simple
1. forked

Character 93

Number of neural arches [New character]

0. equal or more than 50
1. equal or less than 49

Character 94

Posterior neural and haemal spines [Forey 1998: character 90]

0. abutting one another
1. not abutting

Character 95

Occipital neural arches [Forey 1998: character 91]

0. not expanded
1. expanded

Character 96

Ossified ribs [Forey 1998: character 92]

0. absent
1. present

Character 97

Ossified lung [Toriño et al. 2021: character 107]

0. absent
1. present

Character 98

Basal plate of the anterior dorsal fin [Forey 1998: character 101]

0. with smooth ventral margin

1. emarginated and accommodating the tips of adjacent neural spines

Character 99

Fin rays in the anterior dorsal fin (D1) [New definition of character of character 96 of Forey 1998]

0. more or equal to 11

1. less than or equal as 10

Character 100

Anterior dorsal fin [Forey 1998: character 98]

0. without denticles

1. with denticles

Character 101

Basal support of the second dorsal fin [Forey 1998: character 102]

0. simple

1. forked anteriorly

Character 102

Pelvics [Forey 1998: character 100]

0. abdominal

1. thoracic

Character 103

Pelvic bones of each side [Forey 1998: character 108]

0. remain separate

1. fused in midline

Character 104

Diphycercal tail [Forey 1998: character 93]

0. absent

1. present

Character 105

Caudal lobes [Forey 1998: character 97]

0. symmetrical

1. asymmetrical

Character 106

Fin rays [Forey 1998: character 94]

0. more numerous than radials
1. equal in number

Character 107

Fin ray [Forey 1998: character 95]

0. branched
1. unbranched

Character 108

Paired fin rays [New definition of character 99 of Forey 1998]

0. slender
1. expanded

Character 109

Median fin rays [New definition of character 103 of Forey 1998]

0. slender
1. expanded

Character 110

Lateral line openings in scales [Forey 1998: character 105]

0. single
1. multiple

Character 111

Ventral keel scales [Friedman and Coates 2006: character 109]

0. absent
1. present

Character 112

Scale ornament [Forey 1998: character 104]

0. not differentiated
1. differentiated

4.2 Datamatrix

Taxa	Characters 1–112
Onychodus	0 0 0 0 0 - 0 0 - 0 0 0 1 1 0 0 0 0 0 0 0 0 2 0 0 0 1 0 0 1 0 0 0 0 0 0 0 0 0 0 1 - - - - - - 0 1 0 0 0 0 0 0 1 - 0 0 0 - 0 0 1 0 0 0 - - - 0 - 1 0 ? ? 0 1 0 1 - 0 - ? 1 0 0 ? ? ? 0 0 ? ? ? ? ? 1 0 0 ? ? ? 1 ? 0 0 ? ? 1 ? 0
Allenypterus	0 1 ? 0 ? ? 1 1 0 0 ? 0 1 0 ? ? 0 0 0 0 0 1 0 0 0 0 0 0 1 ? 0 0 1 0 1 0 0 0 0 1 1 0 0 0 0 0 0 ? ? ? 1 1 3 0 1 0 0 0 1 0 ? ? ? ? 0 0 0 0 1 ? ? ? ? ? ? ? ? ? ? ? ? ? ? ? ? ? ? 0 ? ? 1 0 0 1 0 0 1 0 0 0 0 0 0 1 1 0 1 0 0 0 1 0
Atacamaia	0 ? ? ? ? ? ? 0 - 1 ? ? 1 0 ? ? 1 1 0 ? ? ? 2 1 ? 0 1 0 ? ? 0 0 1 1 ? ? ? ? ? ? ? 0 1 0 0 ? ? ? ? 0 ? ? ? ? ? 0 0 1 ? 0 ? 0 ?
Axelia	0 0 ? ? ? ? 1 0 ? 0 1 0 1 0 ? 1 1 1 0 2 1 1 0 ? 0 ? 0 ? ? ? ? ? ? ? 0 ? 1 ? 0 ? ? 0 ? ? 0 1 ? 1 ? ? ? ? ? ? ? ? ? 0 ? ? ? ? ? ? ? ? ? ? 0 ? ? ? ? ? ? ? ? ? ? 1 ? ? ? ? ? ? ? 0 ? ? ? 0
Axelrodichthys	0 1 ? 1 1 0 1 1 1 0 1 0 0 0 1 0 1 0 1 1 0 1 2 0 0 1 ? 2 1 0 0 1 0 1 1 0 0 0 0 1 0 1 0 0 ? 1 1 ? 0 0 0 0 1 2 1 0 1 1 1 1 1 0 0 0 1 0 1 1 1 ? 1 0 0 1 1 1 0 0 0 0 1 1 0 ? 1 1 1 0 1 0 1 0 ? 1 1 1 1 0 {01} 1 1 0 0 1 0 1 1 0 0 ? 0 1
Caridosuctor	0 0 0 {01} 0 0 1 1 1 0 ? 1 1 0 ? ? 0 0 0 1 0 1 0 1 0 0 1 0 0 1 0 0 0 0 1 0 ? 0 0 0 1 0 0 0 ? 0 0 ? ? 0 0 0 0 0 0 ? 0 0 0 1 0 0 1 1 ? 0 1 0 0 0 ? 1 0 0 1 ? 0 1 1 0 0 1 0 0 1 1 1 1 0 0 ? 0 0
Chinlea	0 1 ? 0 1 ? 1 1 {01} 0 1 {01} 0 0 ? ? 1 0 1 2 {01} ? ? 1 0 ? ? 2 1 ? 0 0 0 1 1 0 ? 0 0 1 0 1 0 0 0 1 1 ? 0 ? ? 0 ? 2 1 0 ? 1 0 ? 0 ? 1 1 ? ? 1 ? 1 ? ? ? ? ? ? ? ? ? ? ? ? ? ? ? ? ? ? 1 ? ? ? 1 ? ? 1 ? 1 ? ? 1 0 1 0 0 1 ? 1 1 0 0 ? 0 1
Coccoderma	0 0 ? ? 1 1 ? 1 1 0 1 0 1 0 0 1 0 0 0 1 0 1 2 1 0 0 1 3 1 0 0 0 0 0 1 1 ? 1 0 0 0 0 0 0 0 0 ? 1 0 0 1 0 1 3 0 1 ? 0 0 1 0 0 1 0 ? 0 1 0 0 0 ? ? ? ? ? ? ? ? ? ? ? ? ? ? ? ? ? ? 0 ? ? 1 1 0 1 1 0 1 0 {01} 0 1 1 1 1 0 1 1 1 0 1 0 0
Coelacanthus	0 0 0 ? 1 ? ? 1 1 0 1 0 0 0 0 1 1 0 0 1 0 ? ? 1 0 1 ? 3 1 ? 0 0 1 0 1 1 0 ? ? ? ? ? 0 ? ? ? ? 0 ? 0 0 ? 0 ? 1 0 0 0 1 0 0 1 0 0 0 1 0 1 ? 0 ? ? 1 ? 0 1 0 0 1 0 0 0 1 0 0 1 0 1 1 0 0 ? 0 0
Diplocercides	0 0 0 ? ? ? 0 1 1 0 0 1 1 0 0 0 0 0 0 0 0 1 2 0 0 0 1 0 0 1 0 0 0 0 1 0 1 0 0 1 1 0 {01} 0 ? 0 0 0 0 0 0 0 0 0 1 0 0 0 0 0 1 0 0 0 0 0 0 1 0 1 1 1 0 0 0 1 0 1 ? 0 1 0 0 0 0 0 ? ? ? ? ? 0 0 0 ? ? 0 0 ? 0 ? 1 0 0 1 0 0 0 ? 0
Diplurus	0 1 0 0 1 1 1 1 1 0 1 0 0 0 1 0 1 1 0 2 ? 1 0 1 0 1 ? 3 1 1 0 0 1 0 1 1 0 1 0 1 0 1 0 0 0 {12} 1 0 0 ? ? 1 1 3 0 0 1 ? 1 ? 0 ? 0 0 0 1 1 ? ? 1 0 0 ? 1 1 0 0 0 ? ? ? 0 ? 1 1 1 0 1 0 1 0 {01} 1 1 1 0 0 {01} 1 1 0 0 1 0 1 1 0 0 ? 0 1
Dobrogeria	0 ? ? ? ? ? ? ? ? 0 1 ? ? 0 1 1 1 1 0 ? ? 1 ? 1 ? 1 ? 0 1 ? ? ? ? ? ? ? ? ? 0 0 ? 1 1 0 ? ? ? ? ? ? ? ? ? 0 ? ? 1 ? ? ? ? ? ? ? ? ? ? ? 1 0 0 0 1 1 0 0 1 ? ? ? ? ? ? ? 0 ? ? 1 1 0 ? ? 1 ?
Euporosteus	0 0 ? ? ? ? 0 1 1 ? 0 ? 1 ? ? 0 ? ? ? 0 ? 0 ? 0 1 ? ? ? ? 0 1 0 1 0 ? 1 ? 0 ? 0 ?
Foreyia	1 0 0 ? 1 1 1 1 0 0 ? 0 1 0 ? ? ? ? ? ? ? ? 1 4 ? ? ? ? 1 1 0 1 0 1 0 1 ? 0 ? 0 1 1 1 1 0 0 0 1 0 0 ? 0 0 1 1 0 0 ? 1 0 1 0 0 0 1 0 ? 1 0 ? ? ? 0 ? ? ? ? ? ? ? 1 ? ? ? ? ? ? 1 ? ? ? 1 ? ? 1 0 1 1 ? 0 0 ? 0 1 ? 0 ? 1 0 1 1 0 0 ? ? 1
Garnbergia	0 1 ? ? ? ? ? 1 ? ? ? 0 0 0 ? ? 1 ? ? ? ? ? ? ? ? ? ? ? 3 1 ? 0 ? 0 0 1 0 0 ? ? ? 0 ? 0 0 ? ? ? ? ? ? ? ? ? 3 0 0 ? 0 ? 0 1 ? ? ? ? ? ? ? ? 0 ? 0 0
Gavinia	? ? 0 0 0 0 ? ? ? ? ? ? ? ? 1 ? ? ? ? ? ? ? 0 ? ? ? ? ? 0 0 ? 0 0 0 ? ? 0 1 ? ? 0 ? ? ? ? ? ? 0 0 0 0 0 0 0 0 ? 0 0 0 ? ? ? ? ? ? 0 ? 0 1 1 0 ? 0 ? ? 0
Guizhoucoelacanthus	0 1 0 ? ? ? 1 1 0 0 ? 0 1 0 ? ? 1 0 0 1 0 1 2 1 0 0 0 3 1 ? 0 0 0 0 1 0 0 ? 0 1 ? 1 0 0 ? 0 1 ? 0 ? 0 0 ? 3 0 0 ? 0 0 ? 0 ? ? 0 ? ? 1 ? ? 0 ? 1 ? 1 1 0 0 ? 0 1 0 1 0 ? 1 0 1 1 0 0 0 0
Hadronector	0 1 0 0 0 0 0 1 1 0 ? ? 1 0 ? ? 0 0 0 0 0 1 1 0 0 0 1 0 0 1 0 0 0 0 1 0 0 0 0 1 1 0 0 0 ? 0 0 ? 1 ? 0 0 ? 0 0 1 0 0 0 ? 0 ? ? ? ? 0 0 0 0 ? ? ? ? ? ? ? ? ? ? ? ? ? ? ? ? ? ? 1 ? ? 1 0 1 1 1 0 1 0 1 0 1 0 0 1 0 1 1 0 0 ? 0 ?
Heptanema	0 1 0 ? ? ? ? 1 0 0 ? ? ? ? ? ? ? ? ? ? ? ? ? ? ? ? ? ? 3 1 ? ? ? ? ? 1 ? 0 ? 0 ? ? 1 0 ? ? ? ? ? ? ? ? ? ? ? 3 0 0 ? 1 ? 1 0 ? 1 ? 1 1 ? 1 1 1 0 ? 1 0 1 1 0 0 ? ? 1
Holophagus	0 1 0 ? 1 ? 1 1 1 0 1 1 0 0 1 1 1 1 0 ? ? 1 2 1 ? 1 ? 3 1 0 0 0 0 0 1 0 0 0 0 1 1 1 0 0 ? ? {12} ? 1 ? ? 0 1 1 0 0 1 1 0 ? 0 ? 1 1 ? 1 1 0 0 0 ? ? ? ? 1 1 ? ? ? ? ? 1 ? ? 1 ? ? ? ? 1 ? 0 1 1 0 1 0 {01} 1 1 0 0 1 ? 1 1 1 1 ? 0 0
Holopterygius	? ? 0 ? ? ? ? ? ? ? ? ? ? ? ? ? ? ? ? ? ? ? 0 ? 0 0 ? 0 ? ? ? 0 0 ? ? ? ? ? ? ? ? ? ? ? ? 0 0 ? ? ? ? ? ? ? ? ? ? 1 0 0 ? 0 0 0 ? ? ? 0 ? ? 1 1 0 1 ? 0 ? 1 ?
Indocoelacanthus	0 ? ? ? ? ? ? 1 ? 0 ? ? ? 0 ? ? ? ? ? ? ? 1 2 ? ? ? ? 2 1 1 0 ? 0 ? 1 0 0 0 0 1 ? ? 0 ? ? ? ? ? ? ? ? 0 ? 2 ? ? 1 1 1 0 ? 0 ? ? ? ? ? ? ? 1 ? 0 ? ? ? 0 ? ? ? 1 ? ? ? ? 0
Latimeria	0 1 0 1 1 1 1 1 1 1 1 1 0 0 1 1 1 1 0 2 0 1 0 1 1 1 1 3 1 1 1 0 0 0 1 0 0 0 1 1 1 1 0 1 1 2 2 1 1 1 1 1 1 1 0 0 1 1 0 1 0 0 1 1 0 1 1 0 0 0 1 0 0 0 1 1 1 0 1 0 1 1 0 0 1 1 1 1 1 0 1 0 0 1 1 0 0 0 1 1 1 0 0 1 0 1 1 0 0 1 0 0
Laugia	0 0 1 0 ? ? ? 0 ? 0 1 0 1 0 0 1 0 0 0 1 0 1 2 1 0 1 ? 0 1 0 0 0 0 0 1 0 0 ? ? ? 0 0 0 0 0 1 1 0 0 1 0 1 0 0 1 0 0 0 1 0 0 0 0 0 0 1 0 0 0 1 1 0 1 1 0 0 1 0 ? ? 0 0 0 1 1 1 0 0 0 1 0 0 1 1 0 1 0 1 0 1 1 1 1 1 1 1 0 ? 0 0
Libys	0 1 ? ? 1 ? 1 ? ? 0 1 ? 0 0 1 1 1 1 ? ? ? 1 3 1 ? 1 ? 3 1 0 0 0 0 0 1 0 0 0 1 1 0 1 0 1 ? ? ? ? 1 ? 1 2 ? 3 0 1 ? 1 0 1 0 0 1 0 1 1 1 ? 0 0 1 ? ? ? ? ? ? ? ? ? ? ? ? ? ? ? ? ? 1 ? ? 1 0 0 1 1 0 1 0 1 1 1 0 0 1 0 1 1 1 1 0 0
Lochmocercus	0 ? ? ? ? ? ? ? ? 0 ? ? 1 ? ? ? ? ? ? ? ? 1 0 0 0 ? ? ? 0 1 0 0 0 ? ? 0 ? 0 0 ? 1 ? 0 0 ? 0 0 0 1 0 0 0 0 ? 0 1 ? 0 0 0 0 ? 0 0 ? 0 0 ? 0 ? ? 1 0 ? 1 ? ? ? 1 0 0 0 0 1 0 0 1 0 0 ? 0 ?
Lualabaea	0 1 ? ? ? ? ? ? ? 0 ? ? ? 0 ? ? ? 0 1 ? 0 ? ? ? ? ? ? 2 ? ? ? ? ? ? ? ? ? ? ? ? ? ? ? 1 0 ? ? 1 1 ? ? ? ? ? ? 2 0 ? ? 1 1 1 1 ? 0 ? ? ? ? ? ? ? ? ? ? 0 1 ? ? ? ? ? ? ? ? ? ? ? 1
Luopingcoelacanthus	0 ? ? 1 1 ? ? 1 1 0 ? 0 ? 0 1 ? 1 1 0 ? ? ? ? ? ? 1 ? ? 1 ? 0 0 0 ? ? 0 0 0 0 ? 0 ? 1 ? ? ? ? ? ? 0 0 0 1 ? 0 1 1 1 0 1 0 ? ? 0 ? ? 1 ? ? ? 1 0 ? ? ? ? 0 ? 1 ? ? ? ? ? ? ? ? 1 ? ? ? ? 1 0 ? 1 0 0 1 0 0 1 1 0 0 1 0 1 1 0 0 ? 0 1
Macropoma	0 1 1 1 ? 1 1 1 1 0 1 1 0 0 1 1 1 1 0 2 0 1 {02} 1 1 1 ? 1 1 0 1 0 0 0 1 0 0 0 1 1 ? 1 0 1 1 1 1 1 1 0 1 0 1 1 0 0 1 1 0 1 0 0 ? 0 0 1 1 0 0 0 1 0 0 0 1 1 1 0 1 ? 1 1 0 ? 1 1 1 1 ? 1 1 1 0 1 1 0 1 0 1 1 1 0 0 1 0 1 1 0 0 1 0 1
Mawsonia	0 1 ? ? ? ? 1 1 1 0 1 0 0 0 1 0 1 0 1 1 1 1 2 1 0 1 ? 2 1 0 0 1 0 1 1 0 0 0 0 1 0 1 0 1 0 1 1 ? 0 0 0 0 1 2 1 0 1 1 1 1 {01} ? ? 0 {01} ? 1 1 1 ? ? 1 0 ? 1 1 0 ? ? ? ? ? 0 ? ? ? 1 0 ? ? 1 ? 0 1 ? 1 1 {01} 1 {01} 1 0 0 1 0 1 1 0 0 ? ? 1
Megalocoelacanthus	0 1 1 ? 0 1 1 1 1 0 1 ? ? 0 1 1 1 1 0 ? ? 1 3 1 ? ? ? 3 1 ? 2 ? 3 ? ? 1 1 0 1 0 ? 0 0 1 1 1 0 1 ? 1 0 0 1 1 ? 1 0 1 ? ? ? 0 ? 1 ? 1 1 1 1 1 ? ? ? 1 ? ? ? ? ? ? ? ? ? ? ? ? ? ? ? ? ?
Miguashaia	0 0 0 0 0 0 ? 0 ? 0 0 0 ? 1 ? ? 0 0 0 0 0 0 2 0 0 0 0 0 0 ? 0 0 0 0 0 0 1 0 0 0 1 ? 0 ? ? ? ? 0 0 0 0 0 ? 0 0 1 ? 0 0 0 0 ? ? 0 0 ? ? 0 {01} 0 ? 1 ? ? 0 ? ? ? ? 0 0 ? 0 ? 0 1 0 0 0 0 0 0
Parnaibaia	0 1 0 0 ? ? 1 1 0 0 ? 0 0 0 ? ? ? 0 ? 2 1 ? 2 1 ? 0 0 2 1 0 0 0 0 ? 1 0 0 ? ? ? ? 1 0 0 0 1 1 ? ? ? ? ? ? 2 1 0 1 1 1 ? ? ? 0 0 ? 1 1 ? ? ? ? ? ? ? ? 1 ? ? ? ? ? ? ? ? 1 ? ? 0 1 ? ? 1 ? 1 1 0 1 1 ? 0 0 1 0 1 1 0 0 ? ? 1
Piveteauia	0 0 ? ? ? ? ? ? ? 0 1 ? ? 0 1 1 1 ? 0 ? ? ? ? 1 ? ? ? 0 ? 1 ? ? ? ? ? ? 0 0 ? ? 0 ? 0 ? ? 0 ? ? ? 0 1 0 ? ? 0 0 1 1 0 0 1 0 ? ? ? ? 0 ? 1 ? ? ? ? ? ? ? ? ? 0 1 1 ? ? ? ? ? ? ? ? ? ? ? ? 1 ? 0 ? 1 0 1 0 1 0 1 1 ? 1 1 1 0 0 ? 0 0
Polyosteorhynchus	0 1 0 1 0 0 ? 1 1 0 ? 0 1 ? ? 1 0 0 0 0 0 1 1 0 0 ? ? 0 0 1 0 0 0 0 1 0 0 0 0 1 1 0 0 0 ? 0 0 ? 1 ? 0 0 0 0 0 1 0 0 0 1 0 ? 0 0 ? 0 1 0 0 0 ? ? ? ? ? ? ? ? ? ? ? ? ? ? ? ? ? ? ? 0 ? ? 1 ? 0 1 1 0 1 1 1 0 0 0 0 1 0 1 1 0 0 ? 0 0
Rebellatrix	? 1 ? ? ? ? ? ? ? ? ? ? ? ? ? 0 ? 1 0 0 1 1 0 ? 0 0 0 1 0 0 1 0 1 1 0 0 0 ? 0
Reidus	? 1 ? ? ? ? ? ? 0 0 0 0 ? ? ? ? ? ? ? ? ? ? ? ? 1 3 ? ? 1 1 1 0 0 0 0 0 ? 1 1 0 ?
Rhabdoderma	0 0 0 1 0 0 1 1 0 0 1 0 1 0 0 1 0 0 0 1 0 1 0 1 0 0 1 0 0 1 0 0 0 0 1 0 0 0 0 0 1 0 0 0 0 0 0 1 1 0 0 0 0 0 1 0 0 0 1 0 0 1 1 0 0 1 0 0 0 1 1 0 1 0 ? 0 0 0 0 ? 0 0 0 1 0 1 0 ? ? 1 0 {01} 1 0 0 1 1 {01} 0 1 0 0 1 0 1 1 0 0 0 0 0
**Rieppelia**	**1 0 0 ? 0 1 1 1 0 0 1 0 1 0 1 1 0 0 ? 0 0 1 0 0 0 1 ? 1 0 0 0 0 ? 0 1 ? ? 0 0 1 0 ? 1 0 1 2 2 ? ? ? ? 1 1 1 0 0 ? ? ? 1 0 0 0 1 0 ? ? ? ? ? 1 ? 0 ? 1 ? ? 0 1 ? ? ? ? ? ? ? ? ? ? ? ? 1 0 1 1 ? 0 0 ? 0 1 ? 0 0 1 1 0 1 0 0 ? 0 0**
Sassenia	0 0 0 ? ? ? ? 1 ? 0 1 0 1 0 0 1 0 0 0 ? ? 1 2 ? 0 0 1 1 1 1 0 0 0 0 1 0 1 0 0 0 1 0 0 0 0 0 0 1 0 0 0 0 1 0 1 ? 0 0 1 0 ? 1 ? ? 0 1 0 1 ? 0 1 0 1 0 0 ? ? 0 1 ? 0 0 1 1 0 1 0 ? ? 1 ? 0 0
Serenichthys	0 0 0 ? ? 0 ? 1 0 0 ? ? 1 0 ? ? 0 0 0 1 0 ? ? ? ? ? ? 0 0 1 0 0 0 0 1 0 0 0 0 1 1 0 1 0 0 ? ? 0 1 ? 0 0 ? 0 0 1 ? 0 ? 0 0 ? 1 0 ? 1 ? 0 ? ? 1 0 ? 0 ? 1 0 0 1 0 0 ? 0 0
Spermatodus	0 0 0 0 0 0 1 1 1 0 1 0 1 0 0 1 0 1 0 ? 0 1 2 1 0 1 ? 0 0 ? 0 0 0 ? 1 0 1 0 0 0 1 0 0 0 ? 0 0 ? ? ? 0 0 0 0 0 1 0 ? ? 1 0 0 1 1 1 0 ? ? 0 0 1 1 0 ? ? 0 0 1 0 ? ? 0 0 ? 1 0 1 ? 0
Swenzia	0 1 1 ? ? ? ? 1 ? 1 ? ? 0 1 1 1 ? ? ? ? ? ? ? 1 0 1 3 1 1 1 0 0 0 1 0 0 0 1 1 ? 1 0 ? 1 1 1 1 1 0 1 0 1 1 0 0 ? 1 0 1 ? ? ? ? 0 ? 1 0 0 0 ? ? ? ? ? ? ? 1 ? ? ? ? ? ? ? ? ? ? ? ? ? 0 1 ? 0 1 0 ? ? 1 0 ? 1 0 1 ? ? ? ? ? 0
**Ticinepomis**	**0 1 ? 1 ? ? ? 1 0 0 1 ? 1 0 1 1 0 1 ? ? ? ? 2 ? ? ? ? 0 1 ? ? 0 1 ? ? 1 ? 0 0 ? ? 1 1 ? 1 ? ? ? ? ? ? 1 ? 0 0 0 1 {01} 0 1 0 ? ? 1 0 1 1 0 ? ? 1 0 0 0 1 ? 0 0 1 ? ? 1 ? ? 1 1 ? ? ? 0 1 0 1 1 ? 0 0 0 1 1 1 0 0 1 0 1 1 0 0 ? 0 {01}**
Trachymetopon	0 1 ? ? ? ? ? ? ? 0 1 ? ? 0 1 0 1 0 1 1 0 ? ? ? 0 1 ? 2 1 ? ? ? 0 ? ? ? ? ? ? ? ? ? 0 ? ? ? ? ? ? ? ? ? 2 ? 0 1 1 0 1 1 ? ? 0 ? 0 1 0 ? 0 1 1 0 ? 1 ? ? ? ? 0 ? 0 0 ? 1 ? 1 0 ? ? 1 ? 0 1 1 1 1 0 0 1 1 0 0 1 0 1 1 0 0 1 0 1
Undina	0 1 0 ? 0 ? ? 1 1 1 1 1 0 0 1 1 1 1 0 2 ? 1 2 ? ? ? ? 3 1 0 0 0 0 0 ? 0 0 0 0 ? 1 1 ? 0 ? ? ? ? 1 0 1 0 1 0 0 0 1 1 0 ? 0 ? 1 1 0 ? 1 0 0 0 1 0 0 0 1 1 1 0 1 ? ? 1 ? ? 1 ? 1 1 1 1 ? 0 1 0 0 1 ? 0 1 0 1 1 1 0 0 1 0 1 1 {01} {01} 1 0 0
Whiteia	0 1 0 {01} 0 0 1 1 0 0 1 0 1 0 0 1 1 1 0 {12} 0 1 2 1 0 0 1 3 1 1 0 0 0 0 1 0 0 0 0 1 1 1 0 0 0 0 0 1 1 1 0 0 0 0 0 1 1 1 0 1 0 0 1 0 0 ? 1 0 0 0 1 0 0 1 1 ? 0 0 0 ? 1 1 0 1 1 1 1 0 0 ? 1 ? 1 1 0 0 {01} 0 1 {01} 1 0 0 1 0 1 1 0 0 1 0 0
Wimania	? ? ? ? ? ? ? 0 ? ? 1 ? ? 0 0 1 ? ? ? ? ? ? ? ? ? ? ? ? ? 1 ? ? 0 0 ? 1 0 ? 0 0 1 ? 1 0 0 ? ? ? ? ? ? 0 ? ? ? 1 1 ? 0 ? ? ? 1 ? 0 ? ? ? ? ? ? ? 1 ? ? ? ? ? ? ? ? ? ? ? ? ? ? 0 ? ? ? ? ? ? ? ? 0 ? ? ? ? ? ? ? ? ? ? ? ? ? ? 0
Yunnancoelacanthus	0 0 ? ? ? ? 0 1 0 0 ? 0 1 0 1 1 1 0 1 1 0 ? ? ? 1 0 0 1 ? 0 0 0 ? 1 0 0 0 0 0 ? 0 ? ? 0 ? ? ? ? ? ? ? ? 1 0 0 ? ? 0 ? 0 0 ? ? 0 0 ? 1 ? ? ? ? ? ? ? ? 1 0 0 ? ? 1 1 1 0 0 1 ? ? 1 0 0 ? 0 1

Datamatrix. In orange are the corrected scorings.

5 References

1 Cavin, L., Mennecart, B., Obrist, C., Costeur, L. & Furrer, H. Heterochronic evolution explains novel body shape in a Triassic coelacanth from Switzerland. *Scientific Reports* **7**, 13695, doi.org/10.1038/s41598-017-13796-0 (2017).

2 Jarvik, E. Basic structure and evolution of vertebrates, p. i–xvi, 1–575. *Basic structure and evolution of vertebrates* **1** (1980).

3 Forey, P. L. *History of the Coelacanth Fishes*. 419 (Chapman and Hall, 1998).

4 Ferrante, C., Furrer, H., Martini, R. & Cavin, L. Revision of the Middle Triassic coelacanth *Ticinepomis* Rieppel 1980 (Actinistia, Latimeriidae) with paleobiological and paleoecologial considerations. *Swiss Journal of Palaeontology* (in press).

5 Stensiö, E. H. O. On the Devonian Coelacanthids of Germany with Special Reference to the Dermal Skeleton. *Kungliga Svenska Vetenskapsakademiens Handlingar* **16**, 1-56 (1937).

6 Cloutier, R. *The primitive actinistian Miguashaia bureaui Schultze (Sarcopterygii)*. (Verlag Dr. Friedrich Pfeil, 1996).

7 Rieppel, O. A new coelacanth from the Middle Triassic of Monte San Giorgio, Switzerland. *Eclogae Geologicae Helvetiae* **73**, 921-939, doi.org/10.5169/seals-164996 (1980).

8 Cavin, L., Furrer, H. & Obrist, C. New coelacanth material from the Middle Triassic of eastern Switzerland, and comments on the taxic diversity of actinistans. *Swiss Journal of Geosciences* **106**, 161-177, doi.org/10.1007/s00015-013-0143-7 (2013).

9 Cavin, L. & Grădinaru, E. *Dobrogeria aegyssensis*, a new early Spathian (Early Triassic) coelacanth from north Dobrogea (Romania). *Acta Geologica Polonica* **64**, 147-173, doi.org/10.2478/agp-2014-0009 (2014).

10 Millot, J. & Anthony, J. *Anatomie de Latimeria chalumnae. Tome 1. Squelette, muscles et formations de soutien*. Vol. 1. (Ed. du Centre National de la Recherche Scientifique, 1958).

11 Andrews, S., Long, J., Ahlberg, P., Barwick, R. & Campbell, K. Osteology and functional morphology of the sarcopterygian *Onychodus jandemarrai* n. sp. from Gogo, Western Australia. *Transactions of the Royal Society of Edinburgh: Earth Sciences* **176**, 197-307, doi.org/10.1017/S0263593300001309 (2006).

12 Meunier, F. J., Cupello, C. & Clément, G. The skeleton and the mineralized tissues of the living coelacanths. *Bulletin of the Kitakyushu Museum of Natural History and Human History, Series A (Natural History)* **17**, 37-48 (2019).

13 Dutel, H. *et al.* The giant Cretaceous Coelacanth (Actinistia, Sarcopterygii) *Megalocoelacanthus dobiei* Schwimmer, Stewart & Williams, 1994, and its bearing on Latimerioidei interrelationships. *PLoS ONE* **7**, e49911, doi:10.1371/journal.pone.0049911 (2012).

14 Northcutt, R. G. & Bemis, W. E. Cranial nerves of the coelacanth, *Latimeria chalumnae* [Osteichthyes: Sarcopterygii: Actinistia], and comparisons with other craniata. *Brain, Behavior and Evolution* **42**, 1-76 (1993).

15 Millot, J. & Anthony, J. *Anatomie de Latimeria chalumnae. Tome 2. Système nerveux et organes des sens*. (Ed. du Centre National de la Recherche Scientifique, 1965).

16 Wen, W. *et al.* Coelacanths from the Middle Triassic Luoping Biota, Yunnan, South China, with the earliest evidence of ovoviviparity. *Acta Palaeontologica Polonica* **58**, 175-193 (2013).

17 Schaeffer, B. The Triassic coelacanth fish *Diplurus*, with observations on the evolution of the Coelacanthini. *Bulletin of the American Museum of Natural History* **99**, 25-78 (1952).

18 Stensiö, E. A. *Triassic fishes from Spitzbergen*. (Almqvist & Wiksells, 1921).

19 Wendruff, A. J. & Wilson, M. V. H. A fork-tailed coelacanth, *Rebellatrix divaricerca*, gen. et sp. nov. (Actinistia, Rebellatricidae, fam. nov.), from the lower Triassic of Western Gondwana. *Journal of Vertebrate Paleontology* **32**, 499–511, doi.org/10.1080/02724634.2012.657317 (2012).

20 Long, J. A redescription of the lungfish *Eoctenodus* Hills 1929, with reassessment of other Australian records of the genus *Dipterus*, Sedgwick and Murchison 1828. *Records of the Western Australian Museum* **13**, 297-314 (1987).

21 Dutel, h., Herbin, M. & Clément, G. First occurrence of a mawsoniid coelacanth in the Early Jurassic of Europe. *Journal of Vertebrate Paleontology* **35**, e929581-3, doi:10.1080/02724634.2014.929581 (2015).

22 Ørvig, T. A survey of odontodes ('dermal teeth') from developmental, structural, functional, and phyletic points of view. *Problems in vertebrate evolution* (1977).

23 Mutter, R. J. & Heckert, A. B. *Re-investigation of enigmatic fish bones known as colobodontid/perleidid toothplates from the Upper Triassic Chinle Group (southwestern USA)*. (New Mexico Museum of Natural History and Science, 2006).

24 Mondéjar-Fernández, J., Meunier, F. J., Cloutier, R., Clément, G. & Laurin, M. A microanatomical and histological study of the scales of the Devonian sarcopterygian *Miguashaia bureaui* and the evolution of the squamation in coelacanths. *Journal of Anatomy* **239**, 451-478, doi.org/10.1111/joa.13428 (2021).

25 Gross, W. Histologische studien am aussenskelett fossiler agnathen und fische. *Palaeontographica Abteilung A*, 1-60 (1935).

26 Gross, W. Kleine Schuppenkunde. *Neues Jahrbuch für Geologie und Paläontologie, Abhandlungen* **125**, 29-48 (1966).

27 Meunier, F. J., Erdmann, M. V., Fermon, Y. & Caldwell, R. L. Can the comparative study of the morphology and histology of the scales of *Latimeria menadoensis* and *L. chalumnae* (Sarcopterygii: Actinistia, Coelacanthidae) bring new insight on the taxonomy and the biogeography of recent coelacanthids? *Geological Society, London, Special Publications* **295**, 351-360, doi.org/10.1144/SP295.17 (2008).

28 Meinke, D. K. A light and scanning electron microscope study on the dermal skeleton of *Spermatodus* (Pisces: Coelacanthini) and the evolution of the dermal skeleton in coelacanths. *Journal of Paleontology*, 620-630 (1982).

29 Rieppel, O. A second actinistian from the Middle Triassic of Monte San Giorgio, Kt. Tessin, Switzerland. *Eclogae Geologicae Helvetiae* **78**, 707-713, http://doi.org/10.5169/seals-165676 (1985).
